# Shining light on chiral inorganic nanomaterials for biological issues

**DOI:** 10.7150/thno.64511

**Published:** 2021-09-07

**Authors:** Yining Shao, Guilin Yang, Jiaying Lin, Xiaofeng Fan, Yue Guo, Wentao Zhu, Ying Cai, Huiyu Huang, Die Hu, Wei Pang, Yanjun Liu, Yiwen Li, Jiaji Cheng, Xiaoqian Xu

**Affiliations:** 1 Key Laboratory of Cell Biology, Ministry of Public Health and Key Laboratory of Medical Cell Biology, Ministry of Education, China Medical University, Shenyang 110122, China.; 2 School of Materials Science and Engineering, Hubei University, Wuhan 430062, China.; 3 Department of Electrical and Electronic Engineering, Southern University of Science and Technology, Shenzhen, China.

**Keywords:** chiral inorganic nanomaterials, induced optical chirality, phototherapy, neurodegenerative diseases, gene editing

## Abstract

The rapid development of chiral inorganic nanostructures has greatly expanded from intrinsically chiral nanoparticles to more sophisticated assemblies made by organics, metals, semiconductors, and their hybrids. Among them, lots of studies concerning on hybrid complex of chiral molecules with achiral nanoparticles (NPs) and superstructures with chiral configurations were accordingly conducted due to the great advances such as highly enhanced biocompatibility with low cytotoxicity and enhanced penetration and retention capability, programmable surface functionality with engineerable building blocks, and more importantly tunable chirality in a controlled manner, leading to revolutionary designs of new biomaterials for synergistic cancer therapy, control of enantiomeric enzymatic reactions, integration of metabolism and pathology *via* bio-to nano or structural chirality. Herein, in this review our objective is to emphasize current research state and clinical applications of chiral nanomaterials in biological systems with special attentions to chiral metal- or semiconductor-based nanostructures in terms of the basic synthesis, related circular dichroism effects at optical frequencies, mechanisms of induced optical chirality and their performances in biomedical applications such as phototherapy, bio-imaging, neurodegenerative diseases, gene editing, cellular activity and sensing of biomarkers so as to provide insights into this fascinating field for peer researchers.

## Introduction

Chirality is an important biochemical property in biological systems, and widely presents at molecules, cells and tissues level 1. Living systems also show extreme stereospecificity and chirality specificity in uptake, sensing, synthesis, metabolic and other biochemical processing, simply because chirality of biomolecules can determine their binding property with other molecules, introducing remarkable influences on many biological events. Due to the universality and significance of chiral molecules in organisms, fabrication, and application of chiral nanomaterials in the field of biomedicine has attracted widespread attentions within recent decades.

Functional inorganic nanomaterials typically metal and semiconductor nanostructures, as emerging panaceas, have aroused a great deal of interests due to their featured characters of non-invasive penetration capability, multifunctionality, ameliorated bio-compatibility and so forth. However, because of the complexity of cell microenvironments, traditional nanomaterials still endure many challenges and limitations in practical applications such as long-term accumulations/toxicity, bio-chemical stabilities, and post-treatment metastasis/relapse. However, the introduction of chirality can provide new insights for these critical issues: Firstly, nanomaterials with chirality can enhance cellular uptake and prolonged *in vivo* stability in blood, thereby improving therapeutic performances. Secondly, chiral nanomaterials have displayed suitable biocompatibility, for example, the cytotoxicity of chiral carbon nanomaterials can be effectively reduced after chemical functionalization with various surface ligands 2,3. Thirdly, chiral inorganic nanomaterials can express great absorption differences on left-handed polarized (LCP) and right-handed polarized (RCP) radiations, which would help improve the selectivity and accuracy of therapeutic agent activation in tumor regions 2. Furthermore, circularly polarized light (CPL) tailored bench-top synthesis could provide enantioselective effects to the obtained chiral inorganic nanomaterials, *e.g*., twisted or helical morphologies from CdTe crystal growth 4 and chiral assemblies of achiral gold nanoparticles 5.

In general, the substrate materials in many cases provide the basic properties/functions for theranostics. For example, due to the enhanced permeability and retention effect, inorganic nanoparticles can address many challenges that small molecular drugs cannot do. However, they also have troubles with issue about biocompatibility such as long-term stability which leads to accumulation of metal ions in reticuloendothelial systems (RES) such as liver and spleen for long periods of time. Chiral modifications, such as chiral-ligand-based surface functionalization, could to some extent improve these situations due to the encapsulation effect. Moreover, since the whole bio-microenvironment is chiral in life, the chiral NPs can express enantio-selective behaviors particularly in biological systems. For example, NPs with D-ligand can pass the cell membrane more easily than L-ligand protected NPs and bare NPs (See section 5.1 for details). Therefore, chiral structure (let's say achiral NPs + chiral ligands or chiral assemblies of achiral NPs) as an integration often benefits both from the substrates and the chiral elements. More importantly, chirality can as well induce brand-new “properties/functions” that pristine substrate doesn't inherently have. Take our work on chiral MoO_3-x_ NPs for example 6,7, in one way the chiral cysteine ligands induced a circular dichroism effect at NIR range (~800 nm) which is originated from the oxygen deficiency on the surface of MoO_3-x_ substrate material; on the other way, the ligand and metal core interactions generates a strong absorption peak at visible (~580 nm) via the metal-to-ligand charge transfer (MLCT) effect. Such absorption peak with strong chirality belongs neither to the chiral ligands nor to the MoO_3-x_ NPs, it is attributed to the ligand and MoO_3-x_ substrate interactions, indicating sometimes one should not judge chiral structures separately.

In addition, the fascinating properties of chiral structures extend greatly the application area of inorganic nanomaterials. Firstly, properties of the substrate materials can be enhanced. The above mentioned chiral MoO_3-x_ for instance, could show enhanced (~30% enhancement) PTT performance compared to their pristine counterpart due to the chiral effects when irradiated by chiral laser. Moreover, chirality as the nature of nature, can make achiral inorganic materials more bio-friendly and to some extent, apply them into biological administrations. For example, the helical SiO_2_ nanostructures can behave biomimically which in return leads stem cell commitment into osteoblast lineage 8. Similarly, the differentiation of neural stem cells into neurons can be accelerated by circularly polarized photons when DNA-bridged chiral assemblies of gold nanoparticles are entangled with the cells' cytoskeletal fibres 9. In fact, chiral structures can function synergistically. Kuang's group for example, showed that chiral cysteine ligands can recognize and bind to the DNA GATATC fragment. And cysteine protected CdTe NPs can generate reactive oxygen species (ROS) when irradiated, which can be used for site-selective cleavage of such fragment 10 (see Section 4.3 for more chirality involved biological applications).

In sum, based on substrate material's property, chirality actually acts as a role of game changer who often introduces new potentials/properties or enhances the pristine properties/functions of the substrate materials endowing them chances for more and better performances in biological applications, but also new insights for establishing revolutionary strategies for biological issues 11-14. In this review, therefore we focus on current research state and clinical applications of chiral nanomaterials in biological systems with special attentions to chiral metal- or semiconductor-based nanostructures which possess excitonic features. To avoid redundancy, we exclude chiral perovskites, metal organic frameworks (MOFs) and their derivatives which have intuitively differed physicochemical properties, and chiral 2D/3D surfaces which have macroscopic chirality that is far beyond the scope of this review. (For interested readers, a body of extensive cutting-edge reviews about chiral perovskites 15,16, MOFs 17,18 and 2D/3D surfaces 19-21 are published recently concerning the syntheses, mechanisms, and applications.) In section 2, we will present briefly current forms of chirality in metals and semiconductors, followed by a theoretical background introduction to optical absorption and chiroptical responses in these nanomaterials and their endowed optical behaviors such as circular dichroism (CD) and enantioselective photothermal performances (section 3); Section 4 summarizes chiral metal and semiconductor nanostructures based frontiers in biomedical applications in terms of phototherapy, bio-imaging, neurodegenerative diseases, gene editing, cellular activity and sensing of biomarkers; Section 5 will present bio-safety issues which is a pivotal part as the prerequisite for clinic use of chiral inorganic nanomaterials; Section 6 presents a brief summary and potential perspectives of this research area with challenges that remain unsolved to date.

## Chirality in inorganic nanomaterials

**Figure 1:**
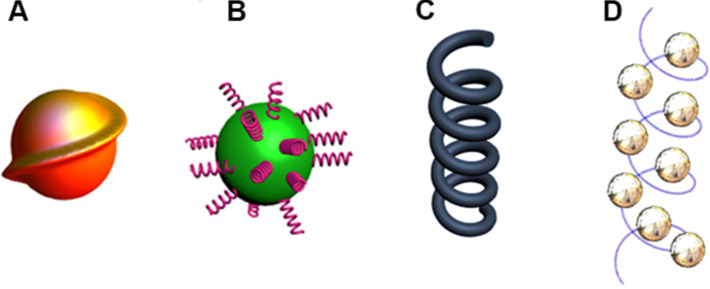
Inorganic nanostructures with chirality. **A.** Intrinsically chiral nanostructure or lattice. **B.** Achiral nanoparticle capped with chiral molecules on the surface. **C.** Nanostructures with chiral shape. **D**. Chiral arrangement of achiral nanoparticles. Adapted with permission from 14, copyright 2020 Wiley-VCH.

**Figure 2:**
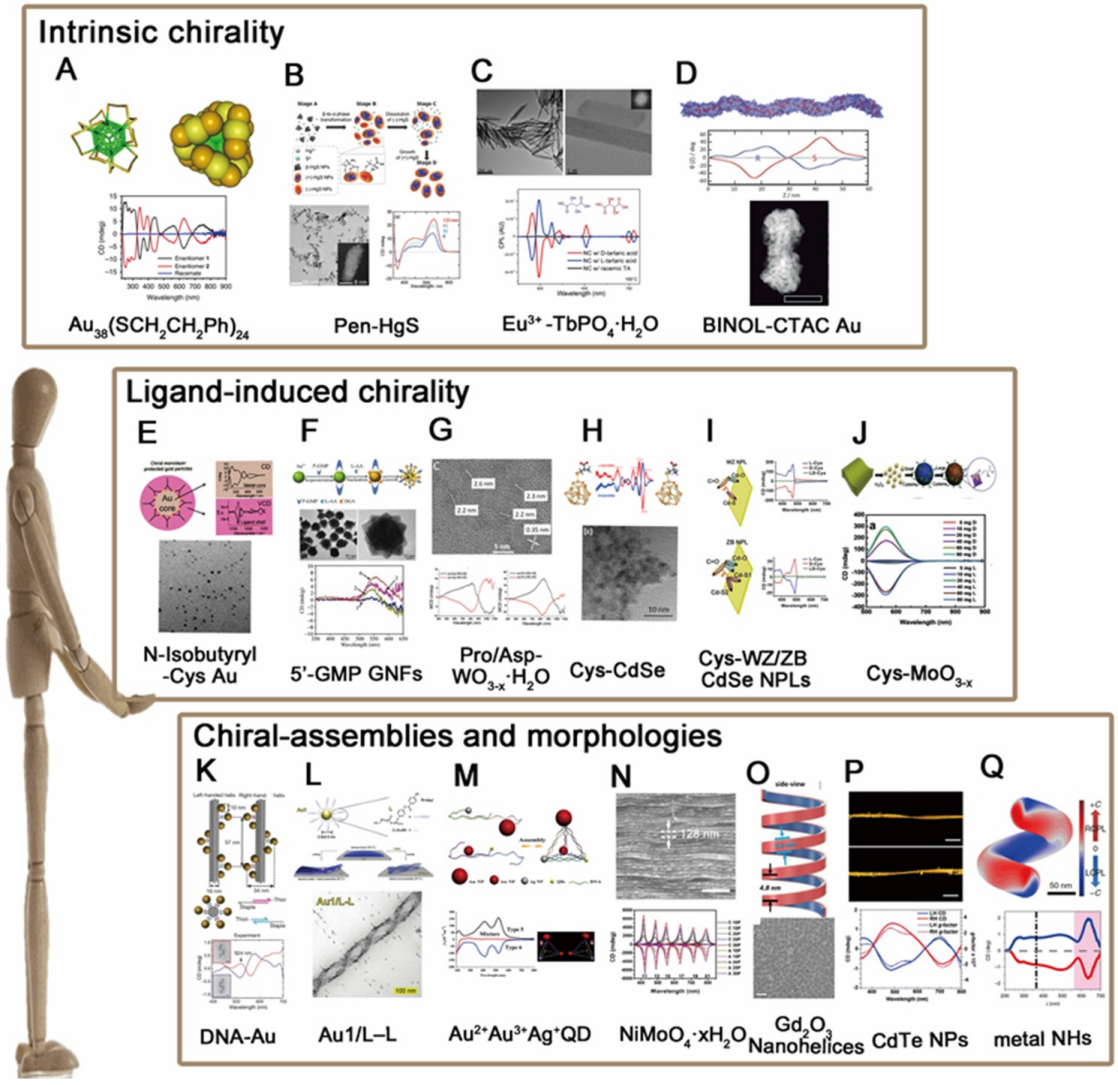
The chiral induction method of nanomaterials. **(A-D)**. Chiral cores **A.** Au_38_(SCH_2_CH_2_Ph)_24_; **B.** Pen-HgS; **C.** Eu^3+^-TbPO_4_∙H_2_O; **D.** CdSe/ZnS QDs; **(E-J)**. Ligand-induced chirality; **E.** N-Isobutyryl-Cys-Au; **F.** 5'-GMP GNFs; **G.** Pro/Asp-WO_3-x_·H_2_O; **H.** Cys-CdSe; **I.** Cys-WZ/ZB CdSe NPLs; **J.** Cys-MoO_3-x_; **(K-P)**. Self-assembly of nanomaterials; **K.** DNA-Au; **L.** Au1/L-L; **M.** Au^2+^Au^3+^Ag^+^ QD; **N.** NiMoO_4_·xH_2_O; **O.** Gd_2_O_3_ Nanohelices; **P.** CdTe NPs. **Q.** chiral Ag NHs **Panel A** is adapted with permission from 24, copyright 2015 Springer Nature. **Panel B** is adapted with permission from 25, copyright 2020 American Chemical Society. **Panel C** is adapted with permission from 26, copyright 2019 Wiley-VCH. **Panel D** is adapted with permission from 27, copyright 2015 American Chemical Society. **Panel E** is adapted with permission from 29, copyright 2006 American Chemical Society. **Panel F** is adapted with permission from 30, copyright 2012 Springer Nature. **Panel G** is adapted with permission from 31, copyright 2017 American Chemical Society. **Panel H** is adapted with permission from 32, copyright 2013 American Chemical Society. **Panel I** is adapted with permission from 33, copyright 2018 American Chemical Society. **Panel J** is adapted with permission from 7, copyright 2018 Wiley-VCH. **Panel K** is adapted with permission from 34, copyright 2012 Springer Nature. **Panel L** is adapted with permission from 38, copyright 2019 Wiley-VCH. **Panel M** is adapted with permission from 41, copyright 2012 American Chemical Society. **Panel N** is adapted with permission from 44, copyright 2019 Wiley-VCH. **Panel O** is adapted with permission from 48, copyright 2020 American Chemical Society. **Panel P** is adapted with permission from 4, copyright 2015 Springer Nature. **Panel Q** is adapted with permission from 50, copyright 2020 Springer Nature.

**Figure 3:**
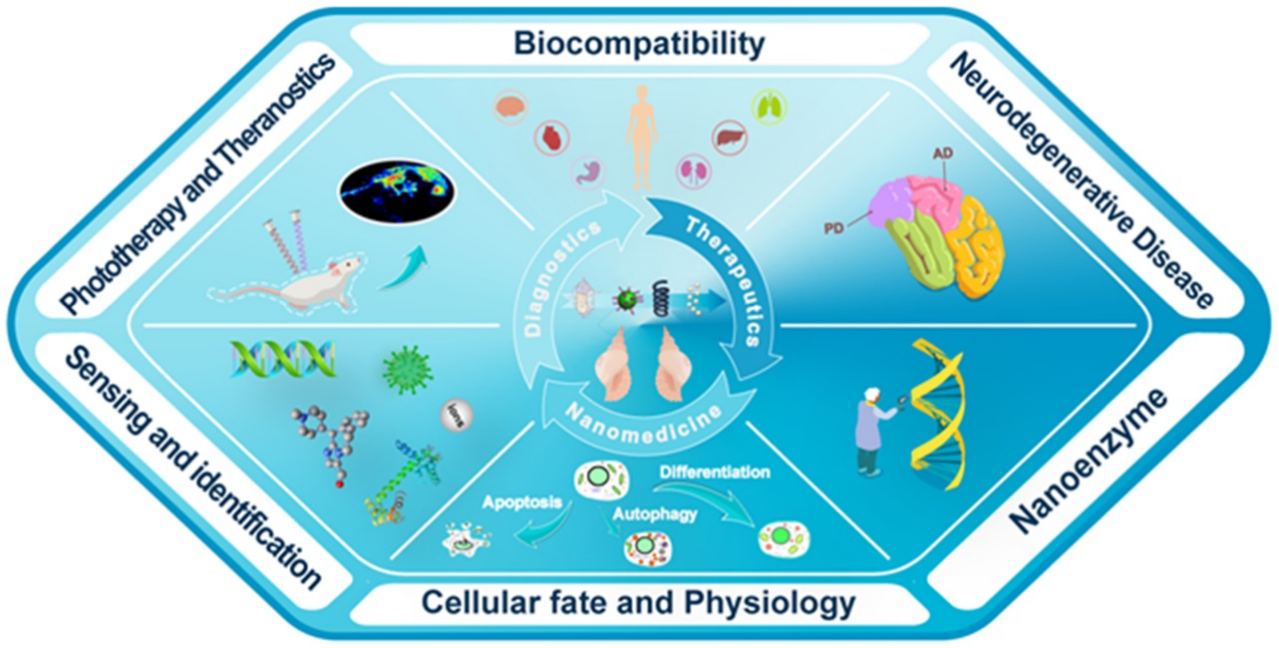
Schematic illustration of biological and clinical application aspects of chiral inorganic nanomaterials.

Compared to chiral molecular systems, chirality of inorganic nanomaterials is a relatively new field. The synthesis of chiral nanomaterials which possess various sizes and morphologies has achieved significant progress during the last decade. In general, chirality in inorganic nanomaterials can be divided into 22: 1) intrinsic chirality formed by chiral lattice distortions and defects (Figure 1A); 2) chiral interactions of achiral NPs with chiral molecules (Figure 1B); 3) chiral shapes with sub-wavelength dimensions or assembled chiral nanostructures of achiral NPs (Figure 1C and 1D). The context below introduces the above mentioned three types of chirality with peer works that frequently appears in recent years, respectively.

### Intrinsic chirality (IC)

The approach inducing chirality in nanomaterials comprises careful design of crystal structures to expose “chiral kinked and stepped surface structures” 23, thereby generating intrinsic chirality of nanostructure. The kink sites lack symmetry, so crystals at the nanoscale can be thought of a chiral pattern, when the step lengths on each side of the kink site are unequal. In this manner, intrinsic chirality has been observed in a bunch of metal and semiconducting nanoclusters or NPs. Bürgi 24 and coworkers, for example, fabricated chiral Au_38_(SR)_24_ nanoclusters (Figure 2A) using thiolate ligands (SR) which showed intrinsically chiral features. Similar intrinsic chirality has also been observed in semiconducting nanomaterials such as HgS nanocrystals 25 (Figure 2B), Eu^3+^ doped TbPO_4_ NPs 26 (Figure 2C) and CdSe/ZnS quantum dots (QDs) 27 (Figure 2D). However, since the synthesis of these nanostructures produces very often racemic mixtures, it is necessary to separate the enantiomers for further applications. So far, Thomas and coworkers have successfully separated the enantiomers of Au nanoclusters induced by achiral thiolates *via* using chiral high-performance liquid chromatography (HPLC) technique. In addition, Fedorov *et al*., demonstrated that chiral CdSe/ZnS QDs can be selectively separated by chiral-ligand assisted phase separation approach in which chiral ligands were applied as separation agents to transfer one enantiomer in aqueous phase and the other in chloroform phase 27. More recently, Sargent and coworkers reported that chirality can be generated from regioselective magnetization of nanostructures. They developed chiral Zn_x_Cd_1-x_S-Ag_2_S/Au@Fe_3_O_4_ combining magnetic component (Fe_3_O_4_) with a series of semiconducting nanorods (NRs), which show selective chirality when induced by a local magnetic field at a specific location 28. Nonetheless, it is worthy to note that intrinsic chirality generally shows weaker CD intensities than that of ligand-induced chirality or chiral assemblies, and it is difficult to discern such type of chirality by high resolution transmission electron microscope (HR-TEM) or CD measurements, which is why the study of intrinsic chirality is still in its infancy and need to be further explored with advanced techniques for stereo-synthesis, chiral separations and theories that explains the formation of chiral motifs.

### Ligand-induced chirality (LIC)

The most common and straightforward approach towards introducing chirality in nanomaterials is to use chiral ligands. For instance, chiral gold nanoparticles (GNPs) can be obtained by adsorption of chiral ligands on the surface such as N-isobutyryl-cysteine (NIBC) 29 (Figure 2E), chiral gold nanoflowers (GNFs) can be prepared using guanosine L-ascorbic acid (L-AA) and 5'-monophosphate (5'GMP) 30 (Figure 2F). In addition to metal nanostructures, semiconductor nanocrystals such as chiral WO_3-x_ NPs 31 (Figure 2G) and CdSe QDs 32 (Figure 2H) or nanoplates (NPLs) with wurtzite (WZ) or zinc blende (ZB) structures 33 (Figure 2I) are also capable for induced chirality simply* via* post-synthetic ligand exchange with chiral thiol ligands (L- and D-cysteine). In fact, apart from capping agents and chirality inducers, chiral molecules can also play as the reducer for the synthesis of chiral inorganic nanomaterials. Tang *et al.*, for example, reported that non-stoichiometric chiral MoO_3-x_ nanocrystals can be obtained *via* fine control over the dose of cysteine (Cys) molecules during the redox reaction of Mo^Ⅵ^ to Mo^Ⅳ^ 7 (Figure 2J). In sum, as the most convenient way to obtain chiral inorganic nanomaterials, ligand-induced chirality has been investigated thoroughly with respects to the properties of core materials (sizes, morphologies, compositions, *etc.*) and chiral ligands (species, conformations, binding modes, *etc.*) respectively. The synthesized chiral NPs have widely applied in interdisciplinary fields such as optical polarizers, chiral spintronics and chirality-based theragnostic devices.

### Chiral assemblies and morphologies (CAM)

As stated in 2.1 and 2.2, chirality in inorganic nanomaterials has been well discovered in atomic and nanometric scales respectively. To deep it further, microscopic chirality is then spontaneously considered as the next research trend in this area. The first explored method is to use biomolecules, DNA, proteins, peptides for example, as the soft templates for chiral assembly of targeted nanoparticles. Among them, one pioneer work is presented by Liedl *et al.*, 2012 in which they reported DNA-based chiral assembly of GNPs *via* using DNA origami as chiral templates and GNPs as building blocks 34 (Figure 2K). Self-assembly chirality can not only be induced by DNA molecules, but also peptides 35,36, such as Au NPs induced by C_18_-(PEP_Au_^M-ox^)_2_ 37. However, chiral nanomaterials induced by chiral peptides are still in the construction stage. And there are no specific biomedical applications.

After that, templating method has been extensively applied to assemble gold/silver NPs. With fine tunability on the sizes of template materials and inorganic NPs, the induced chiroptical activities can be modulated over the full spectrum from ultraviolet (UV) to visible and near infrared (NIR). Moreover, the induced chirality by such method could reach an anisotropic factor (*g*-factor) as high as 10^-1^ level. Similarly, helical assemblies based on the cooperative interactions of liquid crystals (LCs) and GNPs in thin films 38 (Figure 2L) or assembled gold nanorods *via* DNA linkage 39,40, chiral pyramids made from multiple metal and/or semiconductor NPs 41 (Figure 2M), chiral nematic-like films with NPs conjugated with cellulose 42,43 and chiral photonic crystals fabricated by assembly of colloidal inorganic nanowires 44 (Figure 2N) are all successfully demonstrated with possibilities for high value of *g*-factors and tunable chiroptical capabilities in the last decade.

Notably, the involvement of chiral organics is not necessarily a prerequisite for obtaining chiral morphologies or assemblies of inorganic nanomaterials. In 2009, Kotov 45 and coworkers prepared the chiral self-assembly materials by the PCR based on the solid interface of the Au NPs for the first time. Ye and coworkers, for instance, stated that monodisperse nanohelices (NHs) based on gadolinium oxide (Gd_2_O_3_) can be obtained *via* bilayer lattice misfit effect where the continuum elasticity theory of strained bilayers (the 46 and 47 planes of cubic-phase Gd_2_O_3_) accounts for the formation nanohelices 48 (Figure 2O). More interestingly, the chiral transfer of photons to matter provides simplicity and universality for chiral synthesis as well. Kotov and coworkers showed that irradiating a racemic solution of CdTe nanoparticles with left- and right-handed CPL induced the formation of left- and right-handed twisted nanoribbons, with an enantiomeric excess surpassing 30%, yet straight nanoribbons are formed when exposed to linearly polarized light (LP) or in the dark. The author also demonstrated irradiation of the racemic solution with CPL of a specific polarization gave rise to enantio-selective photoactivation of specific chiral nanoparticles and clusters, so that CdTe nanoparticles can self-assemble into nanoribbons with specific helicity 4 (Figure 2P). In a same manner, illumination of gold salt solutions with CPL induced the self-assembly of nanoparticles into chiral nanostructures 10-15 nm in diameter 5. Besides, illuminating NPs with CPL at specific location (nanocuboids corners for example 49) proves as well the possibility to obtain chiroptical responses in gold nanocuboids.

Besides, there are additional kind of chiral nanoparticles fabricated by macroscopic shear force through glancing angle deposition, which are composed of structural chirality at the nano- or atomic- scale. In 2020, Huang and coworkers 50 generated metal NHs, such as L-Ag NHs, R-Ag NHs, L-Cu NHs and R-Cu NHs, through control of the handedness of helical metal nanostructures that are produced by glancing angle deposition onto a substrate that rotates in either a clockwise or counterclockwise direction (Figure 2Q). Chirality induced through glancing angle deposition also observed in chiral Ag NPs 51, chiral Al NPs 52, chiral Ag NPs 53,54, chiral ternary Cu:Au:Pt NPs 55.

In summary, there is no single mechanism for the moment that can comprehensively interpret the origin of chirality in inorganic nanostructures. Different nanomaterials have different chirality inducing methods and chiro-genesis. A variety of different mechanisms may be used to introduce chirality in each of the nanostructures, which may function complementarily or independently. But intentionally or unintentionally, the main purpose of researchers for fabricating such type of nanomaterials is to endow chirality to traditional nanomaterials so as to spring to the forefront of biomedical nanotechnology with chirality-based theragnostic nanomedicines.

## Theoretical background on optical absorption, circular dichroism and enantioselective photothermal performances

To simplify the theoretical background of chiral metals and semiconductors, we particularly employ metal NPs as the representative materials to work with, for which plasmonic theory dominates its absorption behaviors as well as chiroptical responses (while for interested readers in excitonic theory for semiconductors, some related literatures are suggested separately) 56,57. In general, plasmons that could be described as quantum of plasma oscillation are collective oscillation of free electrons gas in noble metals. Similar to general oscillator, they possess their own frequency which is related to the dielectric constant with the presence of external electric fields 58,59. Plasmons manifest as surface plasmon polaritons (or simply called surface plasmons) at the surface of a metal. The oscillating of electric field of incident light would excite surface plasmons resulting standing or propagating plasmon modes in the metal surface. When the size of a nanoparticle is smaller or comparable to the wavelength of light, the free electrons would take part in the collective oscillation if the surface plasmon is confined to the particle. This is termed as localized surface plasmon (LSPR). To understand LSPR in depth, scattering theory is very essential.

When a light is incident into a homogenously conducting spherical particle, the absorption and scattering of the particle could be described by an analytical solution to Maxwell's equation according to Mie theory 60. Representatively, the extinction and scattering cross-section could be expressed as:


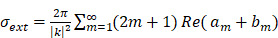
 (1)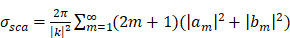 (2)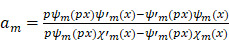 (3)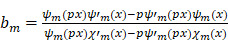 (4)
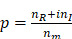

(5)

where 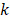 is the wavevector of incident light and 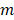 represents the multipoles of the scattering. 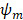 and 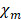 are Riccati-Bessel functions. 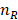 and 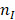 are the real part and imaginary part of refractive index of the metal. 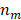 is the refractive index of the surrounding medium. For a spherical nanoparticle, the analytic solution is directly related to the number of multipolar modes 61.

The absorption cross section could be difference between the extinction cross section and scattering cross section. During the light-matter interaction process, plasmon could re-radiate energy, and particles size is a very essential parameter to determine whether scattering or absorption play the dominant role. For large nanoparticles, strong scattering cross-section would be obtained owing to the reduced electron-electron scattering with plasmons' energy re-radiated. Nevertheless, electron-electron scattering could convert the energy of LSPR into heat very quickly, leading to a strong absorption 59.

For particles with size smaller than 10 nm, only the dipole contributes to the plasmon resonances, and the extinction is dominated by absorption. Upon this limit, the absorption cross-section could be described as:


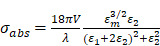
 (6)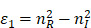 (7)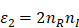 (8)
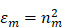

(9)

where 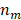 is the volume of the nanoparticle, 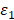 and 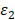 are the real and imaginary parts of the dielectric function of nanoparticle. 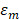 represents the dielectric constant of the medium. It is very easy to be observed that the absorption cross-section would be maximum when the denominator goes minimized. This would be occurred when 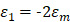. From the formular, the resonance peak would shift longer wavelength with increasing the particle size. In large nanoparticles, the incident light is not total polarizing homogenous which could excite higher order modes in the whole space resulting a red-shift in the spectra 58.

Mie theory is only strictly applied to spherical particles. To investigate optical properties of ellipsoidal particles, Gans theory which is also analytic solutions to Maxell's equation under external electric field influence of ellipsoidal particles with any aspect ratio such as nanorod should be considered 62,63. Similar to spherical particle with size much smaller than wavelength of incident light, the absorption cross-section of an ellipsoidal nanoparticle could be expressed as:


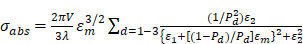
 (10)
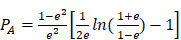
 (11)
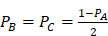
 (12)
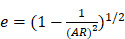

(13)

where 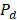 is the depolarized factors of each axis of an ellipsoidal particle, where *B = C < A. AR* represents the aspect ratio of the particle. Considering the absorption cross-section mentioned above, two peaks would be observed from the absorption spectrum. One peak should be addressed to longitudinal resonance corresponding to electron oscillation along the major axis, and another peak comes from electron oscillation across the rod-like particle which is known as transverse mode. The factor weighting of 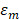 is 2 for a spherical particle. Apparently, the 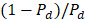 as weighting factor is much larger than 2 in a nanorod with increasing aspect ratio. An increased aspect ratio would lead to a red-shift of plasmon response in the spectrum.

In the limit when particle size much smaller than wavelength, the linewidth of LSPR for both Mie and Gans theory could be calculated as:


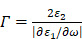

(14)

When the frequency is far from interband transition, the dielectric function is mainly dominated by free electron contribution:


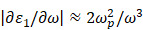

(15)

Where 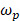 is the plasma frequency. In this case, the linewidth of LSPR is given by:


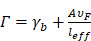

(16)

Here, 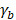 is the damping constant of the bulk metal that is related to the mean free path of electrons. 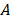 represents a constant that is relevant to electron scattering. 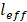 means the effective mean free path length of electrons, and 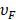 is Femi velocity. Obviously, the linewidth of small nanoparticle for LSPR is directly link to damping of free electron motion by surface and intrinsic electron scattering process. When particle size increases, radiation damping effect among the electron-electron scattering need to be considered. Usually, the radiation damping could be included in linewidth analysis by adding a volume dependent term. Then, the linewidth of LSPR becomes:


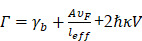

(17)

Here, 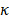 is a constant that depicts the efficiency of radiation damping.

CD spectroscopy is the most widely used tool to investigate chirality of substances including intrinsically chiral molecules, such as amino acid. Comparing with chiral molecule systems, chiral nanostructures has developed for two decades. Usually, chiral nanostructure could be restructured using semiconductor and metal nanocrystals. Chiral molecules play a very important role for constructing chiral nanostructures in many cases. Different mechanisms are suggested for explaining the new CD signals that are observed in experiment.

When nanocrystals conjugated with chiral molecules, the CD effects arise from a chiral atomic structure of a cluster, or from chiral environment. In this case, there are lots of intriguing possibilities. The induction of chirality of the nanocrystals could come from orbital hybridization between the adsorbed molecules and nanocrystals, resulting chirality of electronic surface state of the nanocrystals. Beside this, the chirality could also originate from chiral atomistic defects that are imprinted by the chiral molecules. For very tiny nanocrystals such as clusters, it could have intrinsically chiral atomic structure. From the above, this mechanism is based on nanocrystals with chiral atomistic structure which is similar to pure chiral molecule systems 64-68.

Apart from chiral atomistic structures, the suggested mechanism responsible for CD signals could also be explained by dynamic Coulomb interaction between nanocrystals and chiral molecules 69,70. According to Rosenfeld equation, the rotation strength of chiral molecules is directly related to the imaginary part of product of electric dipole and magnetic dipole transition moment. When consider dipole and multi-Coulomb interaction between molecules and nanocrystals, as summarized by Govorov, the CD activity could be estimated by:


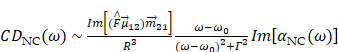

(18)

Here, Im is the imaginary part of the operator, 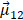 and 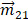 are the quantum matrix of electric and magnetic dipole operators in which the indices 1 and 2 represent the ground states and excited states of molecule, respectively. 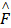 is a dipole orientation matrix. 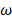 and 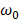 are the frequencies of incident light and transitions of molecules. 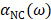
denotes the polarizability of nanocrystals. When consider the influence of nanocrystals, the expression of calculated CD of chiral molecule should be amended:


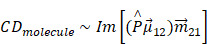

(19)

Where 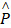 is the electric-field enhancement matrix which describes the changes of electric field with the presence of nanocrystals. This formular means that chiral response of molecules is highly related to the nanocrystals with a dielectric constant which is essentially different from unity.

Chirality could also come from non-chiral metal nanocrystals by reconstructing an optical active system with arranging nanoparticles into chiral geometry such as short helix and asymmetric pyramid 41,71,72. In this case, the CD response that is close to plasmon wavelength could be explained through dipole-dipole plasmon-plasmon interactions. It is evident that the signal is strongly dependent on inter-particles distance and size distribution:


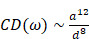

(20)

where 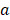 and 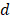 are the radius of metal nanoparticles and inter-particle distance, respectively. When rearrange achiral semiconductor nancrystals into a chiral manner, similar explanation should be acceptable as well. The mechanims of both dynaminc Coulomb interaction and chiral assemblies do not require new chiral atomistic structure, but they involve rearrangment of interactiong non-chiral and chiral nanoscale blocks.

When a non-luminescence nanocrystal is illuminated by a laser, the energy of incident photon would be transformed into heat, and the net increase of temperature can be calculated using 73,74:


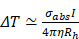

(21)

where 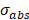 is the absorption cross-section, 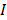 is the intensity of laser, 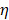 is the thermal conductivity, and 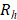 denotes the radius of the heated nanocrystal. If we change the light source into circularly polarized light, the increased temperature of chiral nanostructures would be different under illumination. From this idea, higher conversion efficiency of photothermal effect could be obtained using chiral nanostructures as heater. Generally, the CD from chiral nanostructures is defined as:


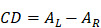

(22)

Here, 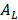 and 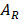 are absorption of nanocrystals incident by left and right polarized light, respectively. To eliminate the influence of nanocrystals concentration and light path length, anisotropy factor is proposed, and which is defined as:


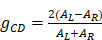

(23)

Similar to CD, the photothermal CD and corresponding anisotropy factor originating from the absorption under circularly polarized light could be defined as:


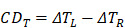
 (24)
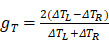

(25)

Here, *∆T_L_* and *∆T_R_* are the increased temperature of chiral nanostructures under laser excitation of left-hand and right-hand circularly polarized light, respectively. Obviously, the anisotropy factor of thermal and optical effect possesses similar tendency since the increased temperature is directly relevant to the absorption cross-section and the intensity of laser excitation.

CPL, on the other hand, reflects the emissive chirality of chiral lumiphores. When a chiral sample is excited with unpolarized light, the difference on photoluminance intensity of generated left- and right- circularly polarized light would be accounted by the CPL spectrometer. By convention, this difference is defined as follows 75:


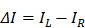

(26)

To quantitatively compare the emission chirality, it is necessary to reevaluate CPL performances by using 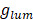-factor like 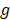-factor for CD measurements which can be defined as 76:


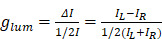

(27)

where 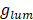 is referred to the luminescence dissymmetry ratio (or factor). Since PL activity depends both on extinction and emission behavior of target materials, quantum efficiency and absorptivity are then critical for understanding the quantity of CPL. For this matter, Zinna *et al.* 77 proposed recently the brightness for CPL, B_CPL_, as a matrix to compare CPL intensity for different materials:


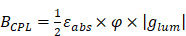

(28)

where *ε*_abs_ and *φ* are the molar absorptivity and the emission quantum yield (QY), respectively.

In summary, with absorption or/and emission dichroism properties, chiral nanomaterials are envisioned with a broad range of practical applications spanning from chiroptics, enantioselective catalysis, spin-polarized devices, to biological issues such as biosensing of biomarkers and polarization-resolved bio-imaging. Since chirality is closely related to the origin of life, the scope of chiral nanomaterials in biological applications should not only focus on the optical phenomena such as above-mentioned CD and CPL properties, more interests can be also applied to human body administrations, metabolism, cell fate, pathology, and neuro diseases with chiral theragnostic nanomedicines. More detailed discussions about applying chirality to biomedical nanotechnology with inorganic theragnostic nanomedicines will be summarized in the following sections.

## Biomedical applications

With the development of chiral fabrication technologies in nanostructure, chiral inorganic nanomaterials guided method have provided a new choice for the diagnosis and treatment of many diseases, and showed great potential for transforming medicine and clinical applications. To elucidate the merits of chiral inorganic nanomaterials in biological and clinical applications, we have collected and arranged current progresses into five aspects, including phototherapy and theranostics, neurodegenerative diseases, cellular fate, and physiology regulations, biosensing and discrimination, as well as gene editing, which were illustrated in Figure 3. In this section, we have focused on these practical results and reviewed the latest achievements, and the chirality generated patterns of the introduced NPs were addressed in the manuscript (CAM represents for Chiral-Assemblies and Morphologies, LIC represents for Ligand Induced Chirality while IC represents for Intrinsic Chirality).

### Phototherapy

Phototherapy is a novel and efficient form of cancer treatment which is light-mediated and non-invasive. The most common phototherapy approaches are photothermal therapy (PTT) and photodynamic therapy (PDT), which can efficiently kill cancer cells through generating selective local heat energy or active oxygen *via* light absorption.

In recent years, chiral inorganic nanomaterials have been developed in tumor phototherapy, as they have shown high efficiency light conversion properties in the window region of biological tissue, as well as exhibited selective absorption properties due to chirality that can reduce indiscriminate injury to normal cells during the therapy. For achieving effective treatment, traditional nanomaterials require either high-power laser, or long-time radiations, which might increase the damage to healthy cells. The introduction of chirality to traditional NPs can provide solutions to these problems. Since the chiral optical activity within nanostructures can be greatly enhanced by the surface plasmon resonance and exciton coupling effects, new materials with super-enhanced properties can be expected by introducing chiral molecules into the traditional inorganic materials.

**Figure 4:**
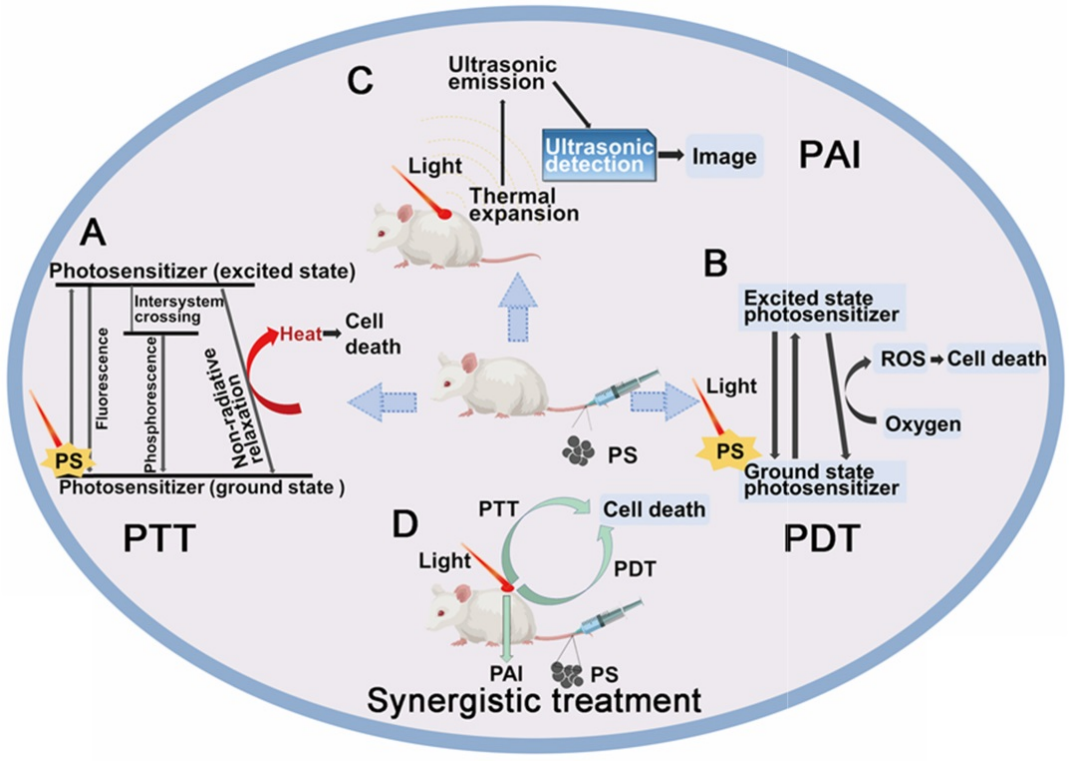
Schematic representation of synergistic diagnosis and treatment of tumor by chiral inorganic nanomaterials. **A.** Mechanism of PTT diagram; **B.** Mechanism of PDT diagram; **C.** Mechanism of photoacoustic imaging (PAI) diagram; **D.** Synergistic diagnosis and treatment of tumor diagram.

For inorganic chiral NPs which functioned as photosensitizers, one common accepted advantage is the enhanced biocompatibility, which has been marked by many works, and also discussed in details in section 5 (Chiral-dependent cellular uptake and bio-safety). The other property is the enhanced phototherapy efficiency and reduced energy consumption. Meanwhile, they have shown as well great potential in photoacoustic effects to generate diagnostic signals to realize the target therapy, the synergistic treatment strategy is shown in Figure 4. In this section 4.1, we will describe the performances and the biological mechanisms of a variety of chiral nanomaterials that can be used for tumor phototherapy.

#### 4.1.1 Photothermal therapy

Photothermal therapy involves a biocompatible agent with large absorption coefficient in the light radiation range. A large amount of photosensitizers (PSs) have been investigated, including organic and inorganic nanoparticles. Compared to the organic dyes, which can be easily degraded by single irradiation, the inorganic metallic nanomaterials can be simulated multiple times to generate heat without degradation. By introduction of chirality, the enhanced biocompatibility and the photothermal conversion efficiency make them the most promising nanomaterials for biomedical applications that can be illustrated by several recent progresses.

**Figure 5:**
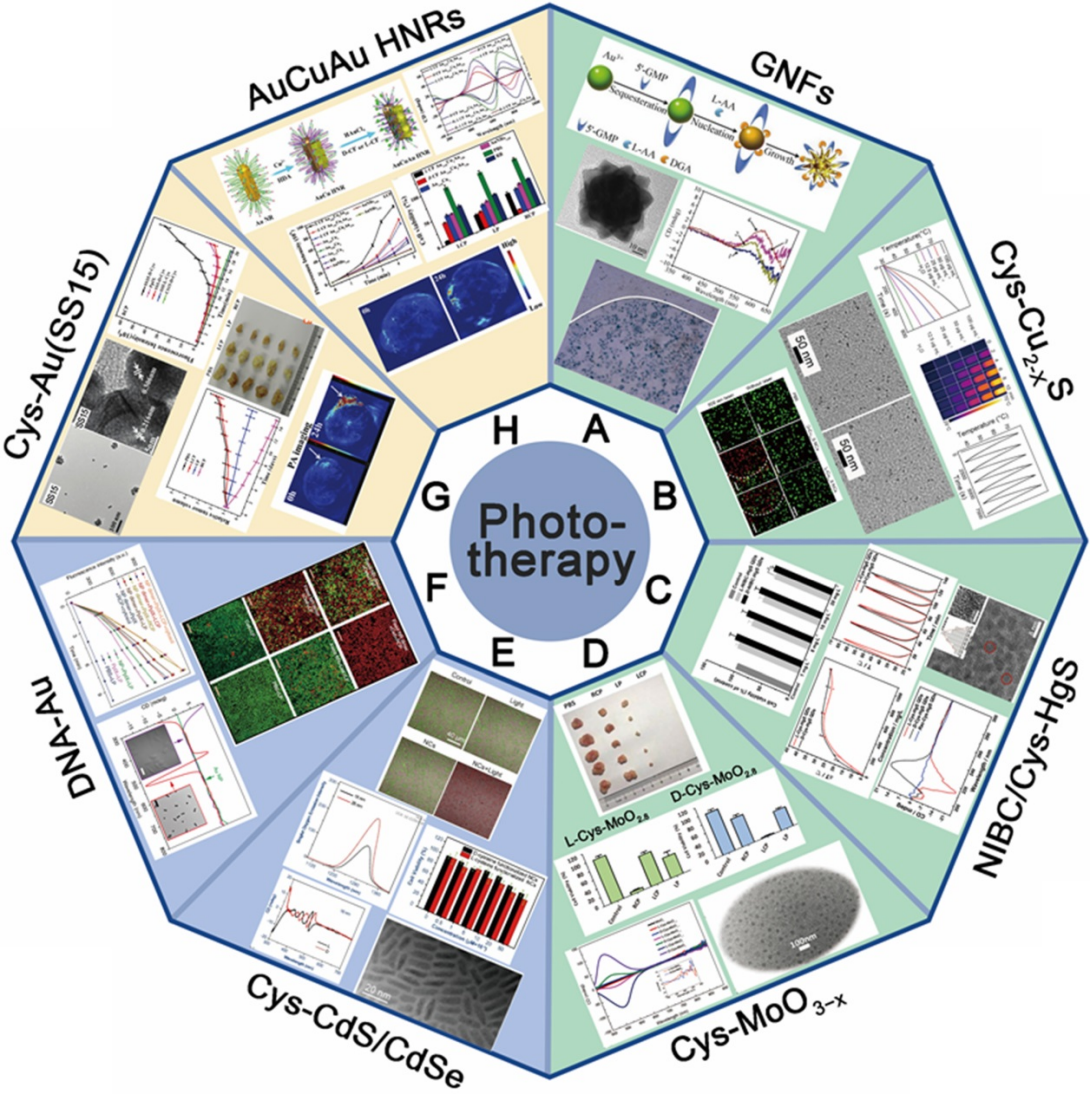
The application of chiral nanomaterials in phototherapy of tumor. **(A-E)** The application in PTT: **A.** GNFs; **B.** optically active Cu_2-x_S nanocrystals (Cys-Cu_2-x_S); **C.** chiral β-HgS QDs; **D.** chiral sub-stoichiometric molybdenum oxide nanomaterials (L-/D-Cys-MoO_3-x_); **(E-F)** The application in PDT: **E.** chiral CdSe/CdS dot/rod NCs; **F.** DNA-Au; **(G-H)** the application in PAI and synergistic diagnosis: **G.** chiral shell-satellites (SSs) gold nanostructures; **H.** chiral alloy AuCuAu HNRs. **Panel A** is adapted with permission from 30, copyright 2012 Springer Nature. **Panel B** is adapted with permission from 78, copyright 2020 the Royal Society of Chemistry. **Panel C** is adapted with permission from 79, copyright 2019 Elsevier. **Panel D** is adapted with permission from 6, copyright 2019 Wiley-VCH and 80, copyright 2021 Elsevier. **Panel E** is adapted with permission from 85, copyright 2019 the Royal Society of Chemistry. **Panel F** is adapted with permission from 86, copyright 2017 Springer Nature. **Panel G** is adapted with permission from 89, copyright 2017 Wiley-VCH. **Panel H** is adapted with permission from 90, copyright 2020 Wiley-VCH.

Chen and colleagues synthesized chiral GNFs with abundant petal-shaped tips using a simple one-pot green synthesis approach in the reduction environment arising from chiral L-AA as a reducing agent and the presence of chiral 5'-GMP 30 (Figure 5A). The size, shape and chirality of the Au-based GNFs (LIC) can be controlled by using different reducing agents and adjusting the reaction time. Biological assays also demonstrated that chiral GNFs have no cyto-toxicity up to 200 μM and exhibited promising biocompatibility with human gastric cancer cell line MGC803. After NIR laser irradiation with a very low energy density of ~30 mW/cm^2^ for 5 min, tumor cells were completely killed, which indicated that chiral GNFs have efficient PTT effect and potential of tumor photothermal-therapeutic agent.

Traditional Cu-based nanostructures were widely employed for phototherapy, once capped with chiral ligands, both of the optical activity and biocompatibility can be enhanced. Xia 78 and coworkers have introduced chiral ligands to NIR optically active Cu_2-x_S nanocrystals (NCs), and D-/L-Cys-Cu_2-x_S NCs have been reliably obtained by ligand exchange (from oleic acid to cysteine) accompanied by the core chemical transformation (from Cu@Cu_2-x_O to Cu_2-x_S) (LIC) (Figure 5B). The D- and L-Cys-Cu_2-x_S NCs have almost identical physical properties such as size, morphology, composition, LSPR band, ligand coating rate, and photothermal stability, except for mirror symmetric CD signals. However, D-Cu_2-x_S NCs exhibited 3 times higher of HepG2 and HeLa tumor cells uptake, as well as the distinctly higher tumor cell ablation efficiency. Without light exposure, both D- and L-Cys-Cu_2-x_S NCs can induce tumor cellular autophagy at a very low concentration (80 μg/mL) by production of ROS, the cells ablation can be further enhanced mainly by photothermal effects guided by Cu_2-x_S NCs under exposure to corresponding CPL. Especially, completely tumors ablation can be efficiently achieved by 40 μg/mL Cu_2-x_S NCs and 10 min phototermal therapy at a power density of 0.75 W/cm^2^. While for normal cells, due to very limited cellular uptake effects, little cytotoxicity has been observed for both D- and L-Cu_2-x_S NCs. In this work, all *in vitro* mechanism of tumor cell ablation were extensively studied, however, the *in vivo* effect was not explored.

In 2019, Sun 79 and coworkers synthesized chiral β-HgS QDs in one-pot by introducing chiral enantiomers N-isobutyryl-L(D)-cysteine and L(D)-cysteine into HgCl_2_ and Na_2_S aqueous solution (LIC) (Figure 5C). By surface modification, chiral β-HgS QDs have obtained the enhanced biocompatibility and selective recognition ability, which provide the biological application possibility for Hg-based materials. In addition, both D- and L-type β-HgS QDs have shown high photothermal conversion abilities, as the *in vitro* irridiation experiments showed that temperature of QDs aqueous solution can reach ~65 °C after exposure under 3 W/cm^2^ 808 nm laser for 5 min, and without degradation after many cycles of heating-up and cooling-down. D-β-HgS QDs also shows better cytocompatibility than its L- counterpart, which is associated with the chirality inversion of chiral β-HgS QDs compared with the corresponding chiral ligands.

In 2019, also by chirality inducing technique, Xu and coworkers have developed L-/D-Cys-MoO_3-x_ (LIC) through the gradual reduction method, exhibiting strong optical activities in both near-infrared and visible light 6 (Figure 5D). *In vitro* tumor cell ablation guided by CPL demonstrated L-/D-Cys-MoO_2.8_ and L-/D-Cys-MoO_2_ nano agents exhibited strong chiral NIR and visible-light sensitivity respectively and high photothermal conversion efficiency. Comparing to classical non-chiral NPs, all the chiral-Cys capped MoO_3-x_ agents exhibited extremely strong chiral effect with low energy consumption under its preferable CPL environment. In 2021, the same group also reported the efficient* in vivo* tumor ablation effect of L-/D-Cys-MoO_3-x_ nano agent guided by PAI 80 (Figure 5D). Meanwhile, they also discovered the related biological mechanism of tumor "delayed effect" during temperature-mediated phototherapy. It is confirmed that photothermal effect caused by L-/D-Cys-MoO_3-x_ NPs can induce delayed cell apoptosis in both intrinsic and extrinsic pathways, which unveils new insights on synergistic relations between photothermal effects and gene expressions. This concept of inorganic theranostic nano-agent qualified with short photo-irradiation time for photothermal therapy provides an efficient and biologically safe approach for cancer treatment which integrates the advances of chiroptics and photo-gene related therapy strategies.

#### Photodynamic therapy

PDT, especially PSs based PDT is another promising phototherapy approach that can be used to treat a variety of tumors 81. In PDT, toxic oxygen species can be produced from PS agents during light exposure, thus eventually causing tumor cells death 82. There are two types of mechanisms known for generating ROS. Type Ⅰ mechanism implicates electron transfer or hydrogen atom abstraction transfer between excited PS drugs and biomolecules, leading to the formation of ROS, such as hydroxyl radicals (·OH), superoxide anion radicals (O_2_·^-^), and hydrogen peroxides (H_2_O_2_). Type Ⅱ mechanism involves energy transfer between excited PS and molecular oxygen in the ground state, thus producing reactive oxygen intermediates (ROI) such as singlet oxygen (^1^O_2_). Compared to type Ⅰ mechanism, ^1^O_2_ can be easily generated with lower light energy, making type II mechanism more important in PDT 83,84.

Compared to traditional PSs, chiral nano PSs have showed the advantages of smaller size, stronger hydrophilicity and targeting, larger specific surface area, and higher bioavailability and surface reaction activity, thus make them the most promising agents in PDT.

Chen and co-workers fabricated two kinds of water-soluble cysteine-capped CdSe/CdS dot/rod NCs (LIC), showing a high photodynamic effect and the potential for PDT 85 (Figure 5E). Chiral cysteine ligands, endowed CdSe/CdS dot/rod nanomaterials with better biocompatibility and water solubility. Due to the defect passivation effect of the surface cysteine ligands, chiral CdSe/CdS NCs exhibited stronger ^1^O_2_ generation capability of 35% in comparison with bare CdSe spherical NCs of about 5% in toluene. By *in vitro* experiments, both D-Cys and L-Cys capped CdSe/CdS dot/rod NCs showed efficient anti-tumor activity under two-photon irradiation.

In 2017, Xu's group fabricated the DNA-bridged NP dimers (CAM) with chiroptical activity exhibiting PDT abilities of malignancies because of the dichroic targeting 86 (Figure 5F). The efficacy of cervical cancer cell elimination was drastically improved when circular polarization of incident photons matched to the preferential absorption of DNA-bridged NP dimers localized inside the cancer cells, which is related to the increased generation of ROS and their preferential intracellular localization. HeLa cells incubated with NP dimers carrying protoporphyrin Ⅸ photosensitizers were killed on a larger scale under 532 nm LCP matched to the preferential absorption of the dimers.

Addition to phototherapy, chiral nanomaterials can also achieve tumor ablation as radiosensitizers or chemodynamic agents. In 2020, Liu 87 and coworkers fabricated an L-Buthionine-sulfoximine (BSO) modified FeS_2_ nanoparticles (BSO-FeS_2_ NPs) which exhibit high photothermal conversion efficiency (49.5%), enhanced generation capability *via* photothermal-improved Fenton effect and photodynamic effect, and good performance on PAI. Chiral BSO-FeS_2_ NPs can achieve PAI-mediated PTT/chemodynamic therapy (CDT)/PDT towards cancer treatment. Besides, BSO-FeS_2_ NPs could activate the repolarization of macrophages from M2 to M1, showing potential for tumor immunotherapy. In 2021, Zhao 88 *et al.* synthesized alkynyl-protected L-/D-Au_10_(C_13_H_17_O_5_)_10_ nanoclusters with excellent radiosensitization effect *in vitro* and* in vivo*, showing the potential for radiotherapy of chiral nanomaterials. In short, chiral nanomaterials can combine chemodynamic therapy and radiotherapy with phototherapy, exhibiting better anti-tumor efficacy and improved safety.

#### Photoacoustic imaging guided phototherapy

Bioimaging and medical imaging aim to reveal the internal structure of the body so as to realize the screening, diagnosis, and treatment of diseases, as well as real-time detection of treatment effects. At present, various imaging techniques have been applied to tumor detection of patients, including ultrasound imaging (USI), magnetic resonance imaging (MRI), X-ray computed tomography (CT) 91. PAI is a novel bioimaging technology developed rapidly in recent years with non-ionizing and noninvasive characteristics, combining the high contrast of optical imaging with the high spatial resolution of ultrasound 92. PAI depends on the photothermal conversion agents converting light energy to heat energy under the laser irradiation, and then converted heat energy causing surrounding tissue to generate thermoelastic expansion, leading to the generation of ultrasound wave. It can be detected with a transducer which can convert the acoustic waves to electric signals. Thus, the image can be formed from the captured signals, providing structural and functional information in preclinical studies, disease diagnosis as well as treatment. Compared with traditional tumor contrast technology, PAI takes the advantage of noninvasive and high contrast 3D optical images with the high resolution and deep tissue penetration. In short, PAI has shown excellent application potential in diagnosis and treatment of cancer and other diseases based on optical materials.

In recent years, with the advances in chiral nanotechnology, chiral nanoparticles-based contrast agents have made significant contributions to photoacoustic imaging of tumor tissues, due to the imaging abilities of deeper tissue and enhanced contrast. In addition, many studies have combined PTT/PDT and PAI to achieve PAI-guided synergistic diagnosis and treatment of tumor based on the optical NPs. Following, this review introduces the application of chiral nanomaterials in photoacoustic imaging and combination therapy.

Xu and colleagues synthesized DNA-driven chiral SSs gold nanostructures (CAM) based on the galvanic replacement reaction, and modified with cysteine enantiomers on nanomaterials surface, making it a promising chiral photosensitizer and imaging agent 89 (Figure 5G). The chiral SS structures displayed great ROS-generating capabilities and could yield more than three times ROS level compared to the classic photosensitizers, such as protoporphyrin Ⅸ(PpⅨ). Both* in vitro* and *in vivo* experiments, chiral SS structures exhibited excellent anti-tumor activity under CPL irradiation. Among various synthesized chiral SS nanostructures, SS15-D-Cys showed the strongest photodynamic effect. After 15 d with RCP light irradiation, the tumor tissue injected with SS15-D-Cys was completely eliminated. Meanwhile, DNA-driven chiral SSs nanostructures were well dispersed throughout the cells, displaying great potential in further clinical application prospect. Besides, chiral SS15-D-Cys exhibited excellent PA imaging capability. The tumor site showed strong PA signal in 24 h after injection, which were clearly identifiable contrasted with the PA signal in 0 h. The result demonstrated that the chiral nanomaterial had the potential to be an PAI contrast agent, making real-time tumor monitoring a reality.

In 2020, Xu *et al.* reported the fabrication of chiral AuCuAu heteronanorods (HNRs) using facile wet-chemistry route and modified with dipeptide cysteine-phenylalanine (Cys-Phe) 90 (Figure 5H). And the optimized chiral AuCuAu HNRs (LIC) with a *g*-factor as high as 0.57×10^-2^ exhibited excellent PTT capabilities. *In vitro* experiments, chiral AuCuAu HNRs also showed ^1^O_2_-producing ability under 808 nm laser irradiation to generate PDT, as well as photostability. *In vivo* experiments, PA signal was detected in tumor tissue abundant with chiral AuCuAu HNRs. Combining PTT, PDT with PAI, chiral AuCuAu HNRs have provided new ideas in the synergistic diagnosis and treatment of tumor and showed great application prospects.

### Neurodegenerative diseases treatment

Metal nanomaterials prepared with chiral amino acids or peptides were reported to have the properties of intense optical activities, good biocompatibility, capability of crossing the blood-brain barrier (BBB) and rather rapid clearance from the body. These properties of chiral metal NPs have attracted significant attentions of researchers, as well as extended their biomedical applicability, such as attempting to inhibit or retard the progression of the neurodegenerative diseases, like Alzheimer's Disease (AD) and Parkinson's Disease (PD) 93-97.

Pathology of the neurodegenerative disorders generally involve aggregation of intra- or extracellular misfolded proteins/peptides 93, oxidative stress caused by ROS 94, and loss of functional neurons. However, current therapies for neurodegenerative diseases are aimed at relieving symptoms but not addressing the underlying pathologies. Nanomaterials can effectively cross the BBB compared with traditional drugs, and the penetration can be facilitated when conjugated with appropriate ligands 95. Several recent works based on chiral NPs are aimed to address the underlying pathology to treat neurodegenerative diseases.

**Figure 6:**
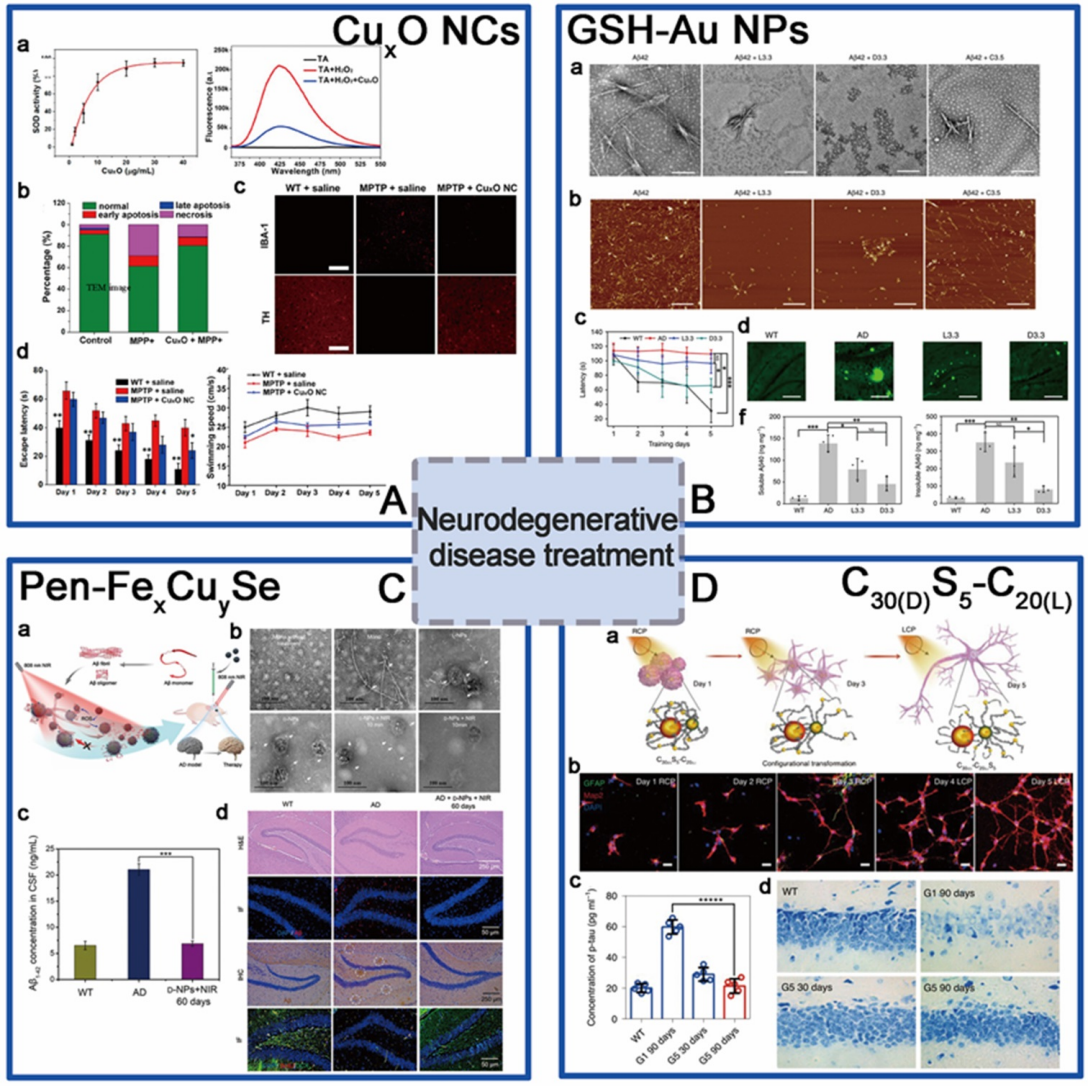
The application of chiral nanomaterials in neurodegenerative disease treatment. **A.** Cu_x_O nanoparticle clusters; **B.** GSH-Au NPs; **C.** Pen-Fe_x_Cu_y_Se; **D.** C_30(D)_S_5_-C_20(L)_. **Panel A** is adapted with permission from 96 copyright 2019 American Chemical Society. **Panel B** is adapted with permission from 97, copyright 2020 Springer Nature. **Panel C** is adapted with permission from 98, copyright 2020 Wiley-VCH. **Panel D** is adapted with permission from 9, copyright 2020 Springer Nature.

For the aim of ameliorating PD, chiral molecule-mediated porous Cu_x_O nanoparticle clusters with an average size of 65±7 nm, have been fabricated, in which Phe as the structure-directing agent 96 (Figure 6A). These Cu_x_O nanoclusters (CAM) can protect cells against oxidative stress, as well as eliminate ROS, thus inhibit the cyto-neurotoxicity, as well as rescue the memory defects in PD model mice. The main functional mechanism is that the chiral Cu_x_O nanoclusters can functionally mimicked the activities of multiple enzymes, such as peroxidase, superoxide dismutase, catalase, and glutathione peroxidase.

Beside scavenging ROS by mimicking antioxidation enzyme activities, chiral metal NPs were also reported that it can inhibit aggregation of amyloid beta (Aβ) fibrillation. In 2020, Tang's group reported a chiral L-and D-glutathione (GSH) modified Au nanoparticles (Au NPs) (LIC) with a diameter of 3.3 nm 97. The smaller size facilitates the penetration of BBB to be more convenient (Figure 6B). Compared with L3.3, D3.3 possesses better inhibition efficiency and protective effect of neurons against Aβ42 aggregates-induced cellular toxicity, as it possesses a larger binding affinity to Aβ42 and higher brain biodistribution. In AD model mice, D3.3 were also more efficient in rescuing memory deficits.

At the same time, Kuang 98 and colleagues synthesized chiral D-/L-Fe_x_Cu_y_Se nanoparticles (LIC) conjugated with D- or L-type penicillamine (Pen), displaying three characteristic CD peaks at 435, 515, and 780 nm (Figure 6C). Under 808 nm near-infrared laser illumination, the NPs can inhibit the self-assembly of Aβ monomers and trigger the dense structures Aβ42 fibrils to become looser monomers by photo-inducing ROS oxidation. D-type NPs were also reported to have higher binding affinities to Aβ42 fibrils than L-type NPs. In addition, *in vivo* experiments also showed that D-Fe_x_Cu_y_Se NPs provided efficient protection against the neuronal damage induced by the deposition of Aβ42 and alleviated the symptoms in a mouse model of AD, leading to the recovery of cognitive competence.

Within the same year, Xu 9 and coworkers reported a DNA-bridged chiral assembly of L-/D-Cys coated gold nanoparticles (CAM), which can accelerate differentiation of neural stem cells (NSCs) into neurons under CPL (Figure 6D). By entangling the cells' cytoskeletal fibres, this Au-based chiral NPs can exert the CPL-dependent force on the cytoskeleton, thus accelerate the differentiation of neural stem cells into neurons. In AD mice, implantation of this CPL-differentiated NSCs can substantially reduce more than 70% of the p-tau and A protein contents, leading to the recovery in their pathologic behaviors.

The applications of chiral NPs in neurodegenerative disorders descend from 2019, until now all the reported works were only focusing on PD and AD, based on Au-based and Au based metal NPs. The main functional mechanism for therapy can be summarized into molecular level as well as cellular level. On molecular levels, chiral Cu_x_O metal NCs can act as multiple enzymes to scavenge ROS; D-Fe_x_Cu_y_Se NPs and D-GSH-Au NPs can inhibit Aβ42 fibrillation through competitive bind to Aβ42 monomers, and both D-type NPs exhibited better performance than L-types in biological systems. On cellular level, L-/D-Cys-Au NPs directly simulate neural stem cells differentiation by exerting the CPL-dependent force on the cytoskeleton of target cells. Based on the functional mechanisms, the biomedical applications of the metal NPs mentioned above can be extended to other neurodegenerative disorders or related cerebral injuries, such as the Huntington's Disease (HD), amyotrophic lateral sclerosis (ALS) and dementia with Lewy bodies (DLB), which were also caused by abnormal aggregation of insoluble proteins and neuronal damages. Furthermore, for all of the above-mentioned nano agents, penetration through the BBB is the critical step for functioning. Therefore, the detailed mechanism of how this chiral nanoagents penetrate the BBB; if the tight junctions of BBB were influenced or destroyed; and how they can be cleaned out from the brain should be further taken into consideration to shorten the gap between theoretical research and the actual clinical applications.

### 4.3 Biomimic nano-enzyme gene editing

Biomimic nano-enzymes have gained much attentions in recent years 99,100, since nanozymes showed the advantages of better catalytic stability, ease of modification, lower cost and longer storage time compared with natural enzymes. However, most of them only functioned to accelerate the chemical reactions. Combination of the chiral properties, chiral nanozymes have demonstrated greater enantioselectivity and sequence selectivity. Recent progress of bio-applications of chiral NPs have exhibited their huge potentials in genome editing and gene therapy, which should attract our attentions.

**Figure 7:**
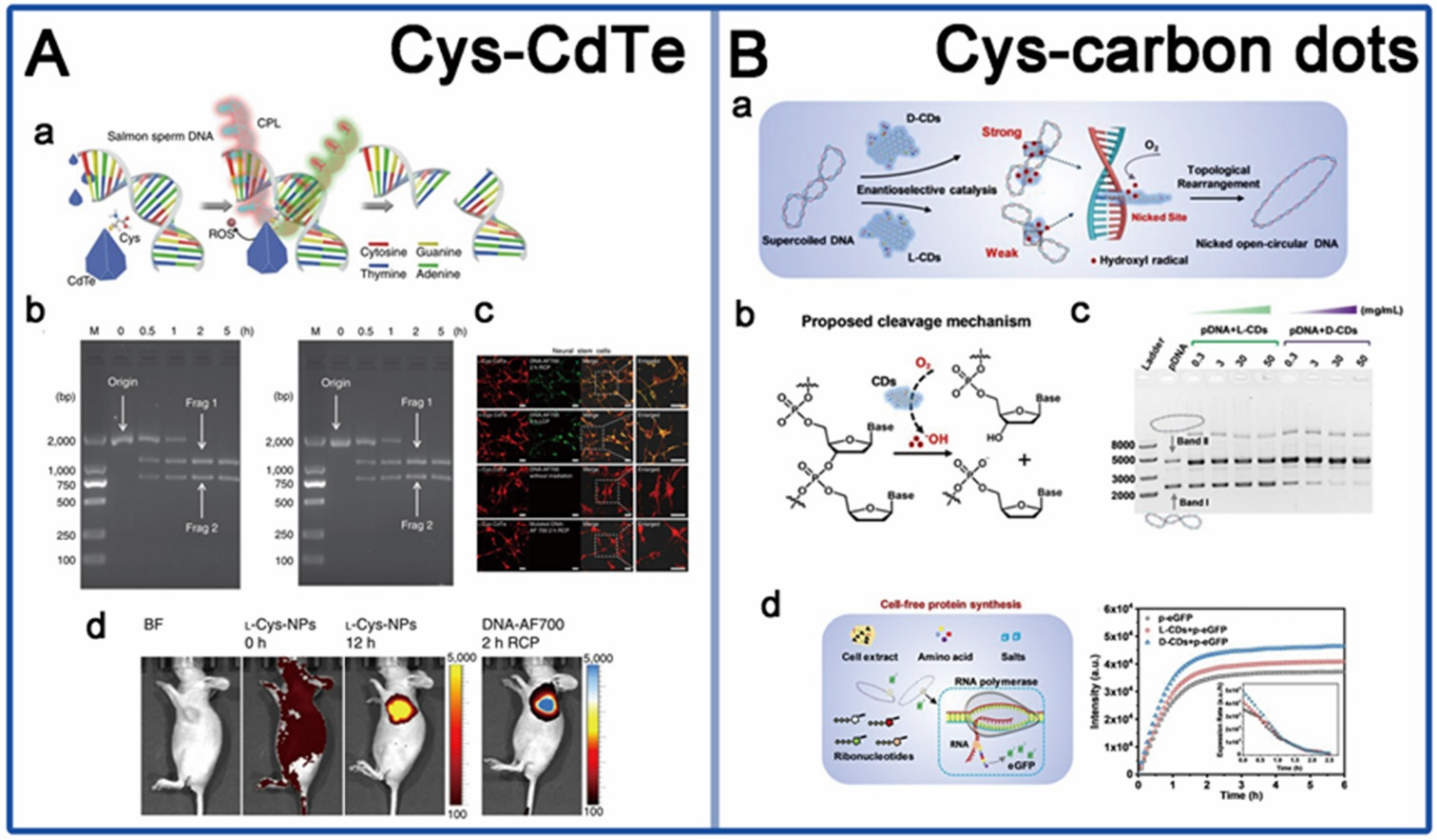
The application of chiral nanomaterials in gene editing.** A.** Cys-CdTe; **B.** Cys-carbon dots. **Panel A** is adapted with permission from 10, copyright 2018 Springer Nature. **Panel B** is adapted with permission from 101, copyright 2020 Wiley-VCH.

Up to now, there are only few reports available in this field, based on mimic engineered nucleases. In 2019, Kuang *et al.* synthesized firstly a water-soluble truncated tetrahedral shape chiral CdTe nanoparticles (LIC) modified with D-/L-Cys, which can specifically recognize and cut double-stranded DNA between the T and A bases at the restriction site GATATC 10 (Figure 7A). Under illumination with CPL at 405 nm, the chiral CdTe nanoparticles can induce the production of ROS leading to cleave the phosphodiester bond of DNA. The special sequence-specific cleavage was derived from the affinity between cysteine and the conformation of the specific base sequence through quantum-chemical calculations. In addition, the chiral nanomaterials showed sequence-specific DNA cleavage activity both *in vitro* and *in vivo*, with high incision efficiency and biocompatibility. CdTe quantum dots were commonly reported for biosensing applications, such as chiral recognition and chiral detection. By the introduction of chiral ligands, the new function of CPL induced DNA phosphodiester bond scission was brought to the CdTe NPs, in addition to sequence recognition, which extends the biological application prospect and value of CdTe NPs.

Another work is from Yang in 2020, they reported a new chiral nanozyme, cysteine-derived chiral carbon dots (CDs) (LIC) 101, which obtained by simply heating the cysteine solution at 80 °C (Figure 7B). This chiral CDs were reported to mimic topoisomerase Ⅰ to mediate the enantiotropic arrangement of DNA superhelices. The underlying mechanism is that intercalative chiral CDs can induce hydroxyl radical generation to cut phosphate backbone in one strand of the DNA double helix, resulting in topological rearrangement of supercoiled DNA. They also found that D-CDs show more effective catalytic activity of plasmid DNA than L-CDs due to strongly intercalative bind with DNA double helix. More innovative materials are still needed, such as virus or bacterial invasion-related protein cleavage and DNA site-specific cleavage. In addition, the current studies were also still lack of precise synthetic strategies to mimic natural active sites for high performance biological interaction. Nevertheless, the development of exploring the innovative applications of the existing chiral assemblies in gene editing should also be promoted.

### Regulation of cellular activity and cell fate

Cell is the basic unit that maintains the basic function of the body. The cells exhibit different capture, endocytosis, and immune responses to the chiral nanomaterials, resulting in the biological processes going in different directions 11,102-105. Studying the interaction mechanisms between chiral nanostructures and biomolecules or cells can help to precisely control their biological activity *in vivo*.

In cell biology and medical applications, it is important to modulate cell-matrix interactions through molecular pathways. Several recent studies based on chiral nanomaterials aimed to explore the mechanisms of their regulation of cellular physiological activities revealing their potential applications in biology and clinical medicine.

**Figure 8:**
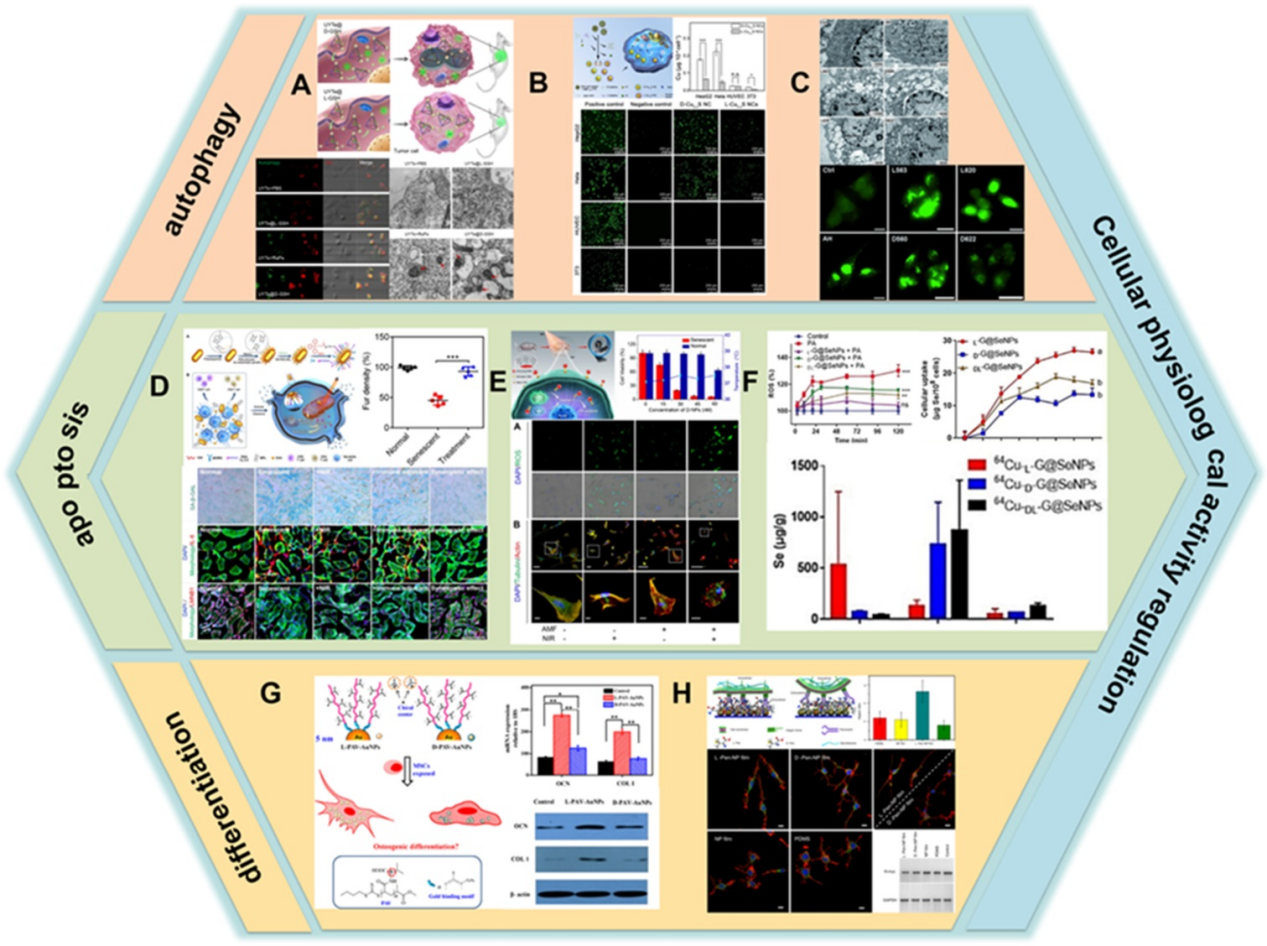
The application of chiral nanomaterials in regulating cellular activity. **A.** GSH-UYTe; **B.** Cys-Cu_2-x_S nanoclusters; **C.** GSH-CdTe QDs; D. aB2MG-TPP@CSNRs; **E.** Pen-Cu_x_Co_y_S NPs; **F.** GSH@Se NPs; **G.** L(D)-PAV-Au NPs; **H.** Pen-Au films. **Panel A** is adapted with permission from 106, copyright 2018 Springer Nature. **Panel B** is adapted with permission from 78, copyright 2020 the Royal Society of Chemistry. **Panel C** is adapted with permission from 107, copyright 2011 Wiley-VCH. **Panel D** is adapted with permission from 108, copyright 2020 Wiley-VCH. **Panel E** is adapted with permission from 109, copyright 2020 Wiley-VCH. **Panel F** is adapted with permission from 110, copyright 2020 Wiley-VCH. **Panel G** is adapted with permission from 111, copyright 2016 Springer Nature. **Panel H** is adapted with permission from 112, copyright 2017 Springer Nature.

The regulation of autophagy by chiral nanomaterials has become a hot research topic in recent years. In 2018, Kuang *et al.* constructed a novel nano-assembly using up-conversion nanoparticles (UCNPs) and yolk-shell nanoparticles (YSNPs) as building blocks to generate UCNP-centered tetrahedral structures using DNA hybridization, and further modified the chirality of the nano-assembly by L-/D-GSH (UYTe) (LIC) 106 (Figure 8A). When it was co-incubated with tumor cells, D-GSH-modified UYTe aggregated in large amounts in living cells while L-GSH-modified UYTe only aggregated in small amounts in endocytic vesicles, and the assemblies exhibited chirality-dependent autophagy-inducing ability. The main mechanism of action is that the accumulated D-GSH-modified UYTe in the cells raise the intracellular oxidative stress and the enzyme-related autophagy activation induces a large amount of ATP production to power the degradation of cellular components.

Additionally, in 2020, Xia's group developed Cu_2-x_S nanocrystals, using L-/D-Cys as the ligand for chiral induction (LIC) 78 (Figure 8B). *In vitro* experimental studies showed that the internalization ability of cells to D- nanoclusters was three times higher than that of L-nanoclusters. Moreover, the uptake ability of nanoclusters by tumor cells is higher than that of normal cells, which is the reason why nanoclusters are significantly more toxic to tumor cells than normal cells. Nanoclusters cause cell death by inducing cell autophagy and its peroxidase activity can convert hydrogen peroxide into hydroxyl radicals in tumor cells, and the production of large amounts of ROS leads to the killing of tumor cells. Besides, the additional photothermal effect of internalized nanoclusters further enhances the ablation ability of tumor cells as described in previous section.

Apart from those mentioned above, Nie *et al*. studied the effects of QDs capped with different chiral forms of the tripeptide GSH on cytotoxicity and induction of autophagy, they fabricated two different sizes of CdTe QDs coated with either L-GSH or D-GSH (LIC) 107. The results showed that QD-induced cell death was associated with an increase in the number of autophagy vesicles (Figure 8C).

Besides inducing autophagy, chiral nanomaterials can also regulate apoptosis in cells. In 2020, Xu *et al.* developed plasmonic core-shell spiky Au nanorods modified with an anti-beta-2-microglobulin (aB2MG) antibody and triphenylphosphonium (TPP) (aB2MG-TPP@CSNRs) (CAM) 108 (Figure 8D). The NRs could work as an immunologic adjuvant to activate dendritic cell, thereby promoting the activation and proliferation of T cells to activate and amplify the host immune response *in vitro* and *in vivo*. Furthermore, under NIR illumination, the NRs were specifically activated to induce the rupture of the mitochondrial membrane in senescent cells through ROS generation and activation of caspase-3 and caspase-7, which induces senescence-selective apoptosis. *In vivo* results showed that the synergistic effect of photo-induced apoptosis and immune adjuvant could efficiently restore the fur density, liver function, and renal function of doxorubicin-induced aging mice.

At the same year, Kuang *et al.* constructed chiral Cu_x_Co_y_S NPs using Pen as a ligand, which can selectively induce apoptosis in senescent cells by generating reactive oxygen radicals and disrupting the cytoskeleton through alternating magnetic field (AMF) and NIR photonic illumination (LIC) 109 (Figure 8E). The synergistic effect increases the efficiency of caspase-3 in activating the apoptotic process. *In vitro* experiments showed that the internalization capacity of D-NPs was 2.5 times higher than that of L-NPs. β-2 macroglobulin-modified D-NPs had a high selective recognition capacity for senescent cells and could effectively remove senescent cells without impairing normal cells activity. *In vivo* studies have shown that D-NPs can successfully remove senescent cells and counteract senescence-induced organ dysfunction through the synergistic effect of photonic light and AMF.

In addition to inducing apoptosis, chiral nanomaterials can also inhibit apoptosis by modulating the expression of intracellular molecules to achieve a protective effect on cells. In 2020, Chen's group prepared chiral Se nanoparticles using phycocyanin as the reducing agent and L-/D-GSH as the chiral ligand (L-/D-G@Se NPs) (LIC) 110 (Figure 8F). The results of research showed that L-NPs were mainly distributed in the liver, kidney, and intestine, while D-/DL-NPs evaded the hepatic metabolic pathway and obtained higher renal clearance. Compared with D-NPs and DL-NPs, insulinoma cells prefer to internalize L-NPs due to the better interaction between L-phospholipid-based cell membranes and L-GSH, and L-NPs inhibit palmitic acid-induced apoptosis by decreasing caspase-8 and caspase-9 activities. In addition, L-NPs can scavenge ROS and prevent mitochondrial damage caused by ROS accumulation, thus achieving cell protection against oxidation.

In addition to the above mentioned, the impact of gold nanostructures on mesenchymal stem cell (MSC) activity has also been investigated. In 2016, Gao *et al.* fabricated chiral poly (acryloyl-L(D)-valine)-anchored gold nanoparticles (L(D) -PAV-Au NPs) and researched the impact on the differentiation fate of MSCs (LIC) 111 (Figure 8G). *In vitro* results showed that the cellular uptake capacity of L-NPs was significantly higher than that of D-NPs, which was one of the reasons for the stronger toxicity of L-NPs to MSCs than D-NPs. Compared with D-NPs, L-NPs could dramatically promote calcium deposition, increase the activity of alkaline phosphatase, and enhance the expression of type Ⅰ collagen and osteocalcin. The regulatory mechanism is that NPs interact with the cell membrane and bind to cytoplasmic proteins, and NPs accumulate in the cytoplasm, leading to mechanical stress on MSCs thereby activating the P38MAPK, ERK1/2 and JNK1/2 pathway, which regulates the expression of related genes to induce osteogenic differentiation and inhibit lipogenic differentiation.

Apart from the aforementioned regulation of cellular activity by chiral nanomaterials, previous studies have shown that chiral nanofilms have certain effects on cell proliferation, differentiation and adhesion. In 2017, Kuang and her colleagues prepared chiral plasma films using Au nanoparticles as the basic building blocks, trisodium citrate as the reducing agent, and L-/D-pen as the chiral ligand (LIC) 112 (Figure 8H). It was shown that most of the adherent cells showed an extended state when grown on L-Pen-NP films, while the cells were predominantly rounded when grown on D-Pen-NP films, what's more, the number of cells grown on L-Pen-NP films was 2.2 times higher than that on D-Pen-NP films at the same time. The cells on L-Pen-NP films differentiated into bipolar neurons, while the cells on D-Pen-NP films differentiated into multipolar neurons. In addition to morphological differentiation, oncoprotein N-Myc expression was also altered. Cells on type L expressed less N-Myc than those on type D.

There is an increasing interest in the study of chiral nanoparticles to regulate cellular behavioral activities, such as the regulation of cellular translocation, metabolism, and aging. Cu_2-x_S 78 generates ROS and induces cellular autophagy, and its additional photothermal effect also enhances cellular ablation; large intracellular accumulation of UYTe 106 increases oxidative stress and activates enzyme-related autophagy to produce large amounts of ATP to power cellular degradation; CSNRs 108 induce apoptosis in senescent cells by activating immune response and generating ROS; Cu_x_Co_y_S 109 induces apoptosis in senescent cells by generating ROS and disrupting the cytoskeleton. G@Se NPs 110 prevent mitochondrial oxidative damage by decreasing caspase-8 and caspase-9 activity and scavenging ROS, thus achieving antioxidant effects. L(D)PAV-Au NPs 111 accumulate in the cytoplasm to increase mechanical stress and thus activate the P38MAPK, ERK1/2 and JNK1/2 pathway, promoting the differentiation of bone marrow mesenchymal stem cells to osteoblasts. The different cell morphology and proliferation behavior on chiral Pen-Au films 112 were due to the different signals released by the stereospecific interactions between fibronectin (FN) and chiral membranes. In conclusion, the regulatory mechanism can be summarized as a joint regulation at the molecular and cellular levels, and ROS plays a very important role in it. Based on the above mechanisms, chiral nanomaterials can be extended to regulate other physiological activities of cells. For example, modulating the immune system of cells and directing the immune system toward a suppressed or activated state provides new therapeutic opportunities for transplant tolerance, autoimmunity, infectious diseases, and cancer treatment. Regulation of cell proliferation, secretion, transport, adhesion, autophagy, apoptosis and other physiological activities may achieve specific cellular functions for diagnostic and therapeutic purposes. However, the above-mentioned NPs all enter the cytoplasm through endocytosis and thus exert their effects. Therefore, it is worthwhile to investigate in depth how to improve the affinity of NPs for cells, reduce their toxic effects on cells, and how to metabolize and excrete them after they have exerted their functions.

### Biochemical and pharmaceutical detection

During the last decades, biosensor and nano-probe application have become popular research topics in chiral bio-nanoscience. A great number of chiral NPs have been fabricated based on the properties of chirality-dependent recognition and discrimination to biological system. The mechanism and sensitivity were mainly determined by the specific-interaction between NPs and ions or biomolecules (including DNA, RNA, proteins, protein secondary structural elements, as well as peptides).

In the physiological environment, highly abundant protein prefers to interact with chiral NPs. Interaction between chiral surface of NPs with the proteins plays an import role in disease diagnosis. Since 2018, researchers have raised their attention to early detection of neurodegenerative diseases, consequently several important works have shown that chiral NPs possess potential application prospects in the diagnosis of neurodegenerative diseases, as the key pathogenic proteins, especially the fibrillation proteins with high molecular weights. For example, D-Fe_x_Cu_y_Se 98 and D-GSH-Au 97 were reported to possesses a larger binding affinity to Aβ42 monomers than L-ones, further irradiated by CPL, both D-NPs also displayed remarkable efficiency in inhibition of Aβ42 monomers aggregation and enhancement on disaggregation of Aβ42 fibrils better than L-NPs. For treating Parkinson's diseases, formation of amyloid fibrils based on a-synuclein can be probed by using Au-plasmonic nanorods 113, which showed no apparent interaction with monomeric proteins but effective adsorption onto fibril structures *via* noncovalent interactions.

Due to the unique size effects and optical properties, metal and semiconductor nanoparticles have also been rapidly developed in a great deal of substance detections related to biomedical application fields, such as food security, pharmaceutical industry, bioengineering, as well as biochemical quota in different organisms.

In this part, we focus on the semiconductor-NPs and noble metal-NPs that can be used in biochemical and pharmaceutical detection fields, as well as discuss their functional mechanisms.

#### Chiral semiconductor-NPs

More attention has been gained by semiconductor compound NPs in recent years due to the relatively lower cost and the diversity of synthetic methods and surface properties. A large amount NPs of semiconductor compounds were QDs. Currently, most metal QDs are composed of Ⅱ-Ⅵ or Ⅲ-Ⅴ group elements, which can be used to contact with chiral molecules to generate chiral metal QDs, with considerable development prospects in biological and pharmaceutical fields.

Generally, chiral metal QDs have unique structures, flexible surface chemical modification capabilities, excellent optical properties, and biocompatibilities, and can achieve high sensitivity and high selectivity for chiral detection.

**Figure 9:**
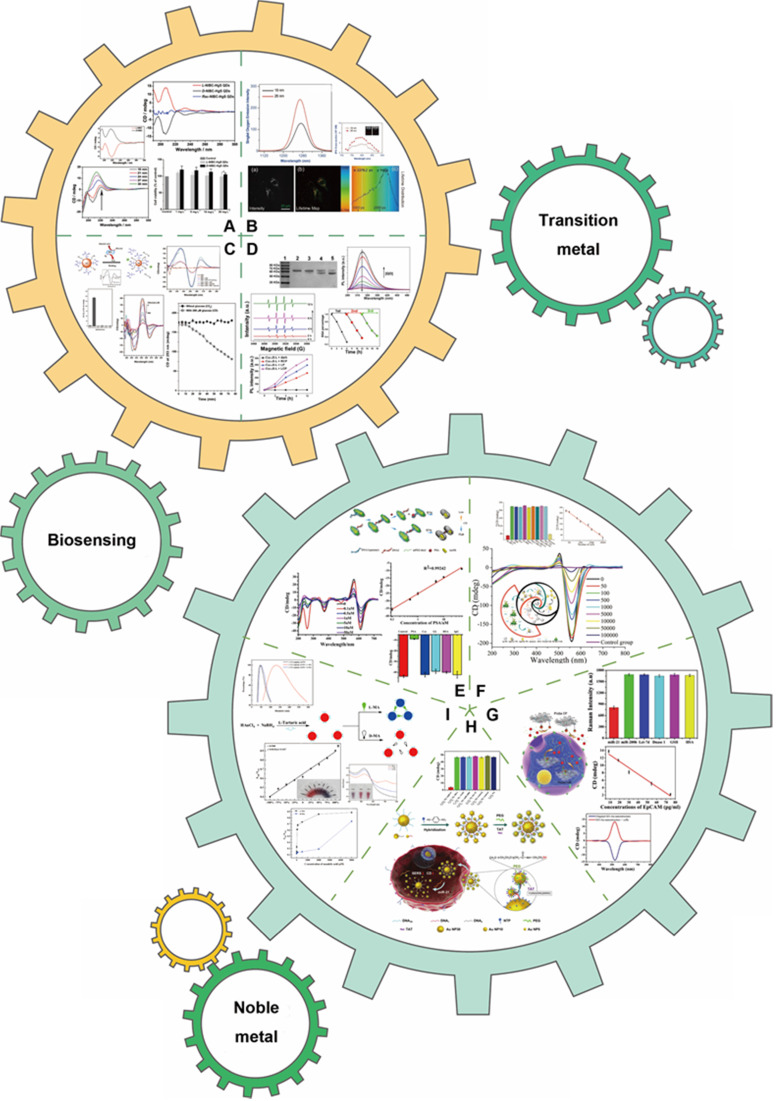
The application of chiral nanomaterials in biosensing.** A.** L(D)-NIBC and L(D)-Cys-β-HgS QDs; **B.** L-Cysteine-functionalized CdSe/CdS dot/rod NCs;** C.** DPA/Cys-CdS QDs; **D.** D-/L-Pen-Cu_2-x_S QDs; **E.** Chirality Au@Ag NR dimers building by aptamer; **F.** Aptamers-driven chiral Ag@Au core-shell NP assemblies; **G.** GO-gold nanoparticle (GO-Au NP) assemblies; **H.** Y-DNA-driven construction of Au NP chiral C_30_S_5_S_10_ NSs; **I.** L-Tartaric acid-capped gold nanoparticles (L-TA-capped Au NPs). **Panel A** is adapted with permission from 79, copyright 2018 Elsevier. **Panel B** is adapted with permission from 85, copyright 2019 the Royal Society of Chemistry. **Panel C** is adapted with permission from 115, copyright 2018 Elsevier. **Panel D** is adapted with permission from 116, copyright 2019 Wiley-VCH. **Panel E** is adapted with permission from 137, copyright 2015 American Chemical Society. **Panel F** is adapted with permission from 138, copyright 2016 Wiley-VCH. **Panel G** is adapted with permission from 139, copyright 2017 Wiley-VCH. **Panel H** is adapted with permission from 140, copyright 2020 Wiley-VCH. **Panel I** is adapted with permission from 141, copyright 2017 the Royal Society of Chemistry.

With the progress of chiral molecule design, chiral metal QDs have overcome their potential toxicity in the field of biomedicine, and exert the greatest advantage of chirality. Baranov *et al.* 114 reported the study of L-/D-Cys-terminated cadmium selenide/zinc sulfide quantum dots (CdSe/ZnS QDs), and investigated the effect of the chirality of the QDs on the uptake efficiency of Ehrlich ascites cancer cells. In 2019, Sun *et al.* 79 introduced the enantiomers (D)-NIBC and L-/D-Cys into aqueous solutions of mercury chloride and sodium sulfide Synthesis of chiral-mercury sulfide quantum dots (HgS QDs) (LIC) (Figure 9A). The study found that the chirality came from the asymmetric arrangement of chiral ligands on the surface of the achiral core, and the biocompatibility of the material was systematically investigated. The data showed that the cell compatibility of D-β-HgS QDs (LIC) shown none-toxic even at high concentrations (20 mg/mL) has better correspondence than L-β-HgS QDs, therefore they can be used as materiel for near-infrared fluorescent probe for biomedicine in organism. In 2019, Chen and co-authors have also fabricated a water-soluable chiral CdSe/CdS dot/rod nanocrystals 85. The chirality of the dot/rod NCs were generated by chiral-cysteine molecules, they also preserve high fluorescence quantum yield, longtime, and efficient CD (Figure 9B). Furthermore, the dot/rod NCs exhibit a high singlet oxygen generation efficiency of 35% meanwhile produce low cytotoxicity, based on which mechanism, it can be used for multiphoton-excited photodynamic therapy on cancer cells. The experimental results also confirm that this water-soluble cysteine-capped CdSe/CdS dot/rod NCs (LIC) are promising materials for applications involving two-photon fluorescence lifetime imaging and photodynamic therapy.

Other than cancer therapy, Cd-based QDs have also gained broad biomedical applications in many other fields, such as blood glucose detection. 2018, Ngeontae's group 115 have reported a chiral CdS QD, which can be prepared by simple mixing cysteamine-capped CdS QDs (Cys-CdS QDs; achiral QDs) with D-penicillamine (DPA) (LIC) (Figure 9C). The as-prepared DPA/Cys-CdS QDs are active in CD spectroscopy due to the chirality of DPA. The principle of glucose detection is based on the destruction of chiral QDs by the H_2_O_2_ generated *in situ* from the enzymatic reaction of glucose oxidase (GO_x_) and glucose in the presence of dissolved oxygen. Comparing to other merits of glucose sensors fabricated from other sensing platforms, this developed Cd-based QDs exhibited excellent selectivity and sensitivity, which have great application potentials.

Some chiral nanoparticles with lattice deformation can be fabricated as well with adjustable chiral properties, such as copper sulfide (Cu_2-x_S), HgS and CdS,* etc.* Among them, Cu_2-x_S nanocrystal is a *p*-type semiconductor material, which has broad application prospects in the field of biomedicine. Kuang *et al.* 116 used L-/D-penicillamine as the chiral precursor and synthesized chiral Cu_2-x_S QDs (LIC) whose anisotropy coefficient was up to 0.01 with excellent photocatalytic activity under CPL radiation, which could affect bovine serum cleavage (Figure 9D). The study indicated the potential chiral applications of copper sulfide nanomaterials in biosensing and medicine fields by combining CPL with chiral QDs to trigger light-induced proteolysis for the first time. Numerous other chiral semiconductor metal NPs which can be applied in biochemical and pharmaceutical detections were summarized in Table 1.

#### Chiral noble metal NPs

Nobel metals mainly refer to gold, silver, and platinum group metals (Au, Ag, Hg, Pt, Pd, Ir, Rh, Ru and Os). Considering the diversity properties, noble metal nanomaterials can be assembled into chiral structures, and show CD spectroscopy response. Generally, optical activity can be enhanced by chiral assemblies with chiral synergistic effect (CSE). For example, the CD signal of a single chiral gold-based nanocluster is relatively weak, while the self-assembled gold nanoclusters have local enhanced electromagnetic fields, which can possibly enhance the chiral signal and be applied to the detection of the trace analysis based on chiral-sensor.

As the detector of biological indicators of some diseases, such as bio-macromolecules, some noble metal chiral materials have shown attractive application prospects. In 2015, Xu 137 and the co-authors fabricated a new gold nanorod (Au-NR) dimer assembly (CAM) that can be used for ultra-sensitive detection of prostate-specific antigen (PSA) (Figure 9E), the specific indicator of prostate inflammation and prostate cancer. In absent with PSA, Au-NR-DNA1 and Au-NR-DNA2 were hybridized to form a dimer probe. With the addition of PSA, the aptamer was more inclined to change its structure and combine with PSA, thereby dehybridizing the dimer to form a single Au-NR. Briefly, CD intensity was weaker under the higher PSA concentration, which can enable ultra-sensitive PSA detection. In addition, the material had also achieved satisfactory test results in actual blood samples.

Tumor cells discrimination, can also be realized by recognition of the bio-markers using aptamer attached chiral NP assemblies. A chiral Ag@Au core-shell NP assembly (CAM) was reported by Song's group in 2016 138, that can be used for special discrimination of the circulating tumor cells in living organisms (Figure 9F). The main function mechanism is based on the sensitive affinity between the aptamers with the specific markers HER2 from the breast cancer cells. To evaluate the specificity of separating cancer cells with HER2, ten types of cells and their mixtures were tested by this NP assembly. Final CD signal exhibited a significant reduction of SK-BR-3 cells (with HER2) and the mixture which contains SK-BR-3 cells, which ascribed to the unique bio-affinity between the aptamers and the HER2 overexpressed on SK-BR-3 cells and the stable chiral plasmonic sensors fabricated by the Ag@Au NP assemblies. The results indicate that the chiral Ag@Au NP assemblies have demonstrated an exceptional selectivity and sensitivity for monitoring cancer cells under circulating tumor cells (CTC) environment, and are robust and stable enough to withstand interference from the multiple peptides and proteins present in blood, which is promising for clinical applications.

Based on the principle of multiple signals generation, the potential perception ability of two kinds of signals can be extended to the detection of two kinds of target substances, such as two different biomarkers. In 2017, Xu *et al.* 139 reported a novel photoelectric material, graphene oxide (GO)-gold nanoparticle (Au NP) assembly (CAM) (Figure 9G). This NP assembly was designed with two regions at both ends that are complementary with the DNA sequence anchored on the surface of the GO and the Au NPs, which can detect microRNA (MIR-21) and epithelial cell adhesion molecules (EpCAM). They also reported that hybridization between miR-21 and NP probe resulted in the separation of 6-fluorescein-phosphoramidite modified Au NPs from GO hence reducing Raman signal. Meanwhile, CD intensity gradual weakening is also shown in the recognition process of EpCAM. Combining the unique electronic properties of GO and Au NPs, the chiral biosensor can detect Mir-21 and EpCAM with high selectivity. The linear ranges were respectively 8.47~74.78 pg/mL, 0.07~13.68 amol/ng RNA, and the detection limits were 3.63 pg/mL and 0.03 amol/ng RNA. The above results were in good agreement with the results of Raman spectroscopy and confocal biological imaging, which opened a new way for highly sensitive detection of clinical diseases. Due to the irreplaceable role chirality playing in biological recognition, the development prospects of metal nanoparticles with chiral optical activity in biosensing and stereoselective reaction have been broadened.

Similarly, for biomarker detections, Kuang's group 140 has also reported a chiral Au-based double-layer core-satellite nanostructure, which is construct by the self-assembly strategy and based on Y-DNA hybridization (CAM) (Figure 9H). The nanostructure (C_30_S_5_S_10_ NS) was constructed by using 30 nm gold nanoparticles (Au NPs) as the core, 5 nm Au NPs as the first satellite layer, and 10 nm Au NPs as the second satellite layer, resulting in very strong CD signals and surface-enhanced Raman scattering. As the cellular biomarkers, specific miRNA molecules that carried by different cell types can be detected by hybridization with these nanoprobes and can be distinguished by causing the drop in the CD and Raman signals.

In addition, numerous chiral NPs were designed and applied in the selective recognition of chiral drug enantiomers, which also created new opportunities for the development of biosensors. For example, Li *et al.* 141 have carried out the visual detection of glutamine (Gln) enantiomers with Au-NRs (LIC) as colorimetric probe (Figure 9I). Many other chiral sensors based on noble metal NPs were also summarized in Table 2. The range of analyte concentrations and the LOD are given where available.

## Chiral-dependent cellular uptake and bio-safety

Natural biological system displays high selectivity for chiral molecules. With the rapid progress of nanobiomedical field, chirality-dependent interactions between nanomaterials and biological system (including cells and bio-molecules) have emerged as a foundation and safety issue for evaluating their biomedical application prospect. Chen and co-authors have summarized the stereospecific interactions between chiral inorganic nanomaterials and different biological systems 11, in this section, we focus on discussing the chirality-dependent biological toxicity based on the latest work and progress, which can be divided into the process of cellular uptake and after internalization into the cells.

### Chiral-dependent cellular uptake

Nanomaterials entering cell is the primary step for performing their function, which can be determined by both cell type and the physiochemical properties of the nanomaterials. Generally, phagocytes (such as monocytes, macrophages, and neutrophils) can take the large particles up to 5 μm through phagocytosis and macropinocytosis, while other cell types only take the particle through different endocytosis pathways.

**Figure 10:**
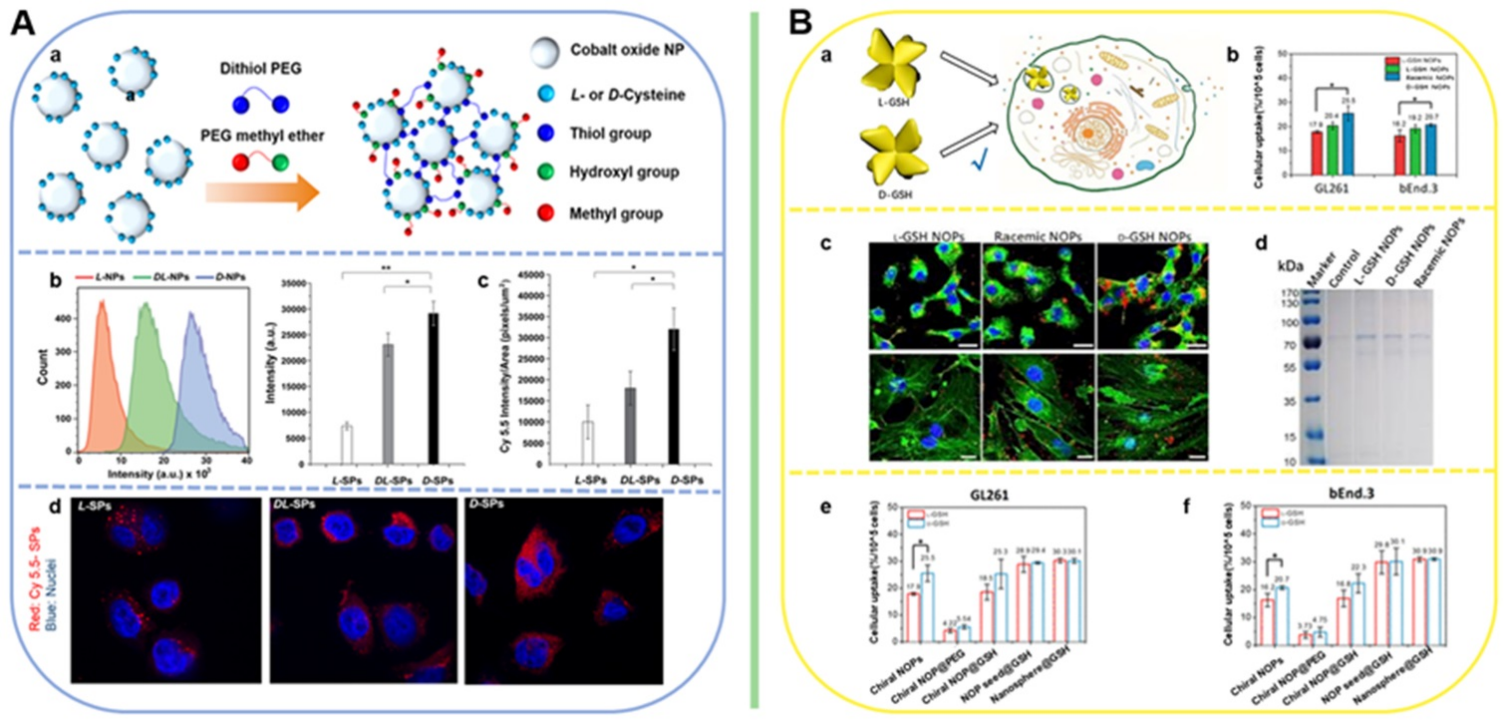
The cellular uptake and cytotoxicity.** A.** Cys-cobalt oxide SPs. **B.** GSH-Au nanooctopods. **Panel A** is adapted with permission from 185, copyright 2019 WILEY-VCH. **Panel B** is adapted with permission from 186, copyright 2021 Chinese Chemical Society.

Despite the cell type, the physiochemical properties (generally particle size and surface charge) of the nanomaterials play critical roles in regulating the process of cellular internalization, distribution in organelles or among organs, and penetration in tumor tissues, and so forth 184. As mentioned above, particle size is the main factor of the pathway that determine how they enter cells. For inorganic NPs, within 2-100 nm were the preferable size range for clathrin- and caveolae-independent endocytosis. For surface charge properties (cationic, neutral, or anionic), positively charged and neutral NPs are more accessible to cells, as the cell membranes are negatively charged. Thus, they are widely used as adjuvants for pharmaceutically active cargos. However, cationic nanomaterials have severe cytotoxicity and be eliminated rapidly from blood plasma, which are significant challenges towards their clinical translation. Recently, Jiang and co-workers have reported a method that can improve the biosafety and pharmacokinetics of cationic gold nanoclusters (GNCs) by substituting the L-GSH with its D-counterpart 184. Compared with L-GSH, modification by D-GSH on the surface can obviously depress their cytotoxicity, hemolysis, or acute damage to organs, which might be due to the less cell internalization of cationic D-GNCs. Similarly, Jaklenec has fabricated a kind of cobalt oxide based chiral NPs and coated by D- and L-Cys, and D-Cys engineered assemblies exhibit the enhanced stability and prolonged biological half-lives* in vivo* than L- ones (Figure 10A) 185. Liu *et al.* has also investigated the chiral-dependent cellular uptake efficacy and biological effects of Au-based nanooctopods (NOPs), results showed that D-GSH NOPs demonstrate greater than 30% enhanced cellular uptake in both GL261 and bEnd.3 cells compared with L-GSH NOPs (racemic NOPs), which could be attributed to the higher adhesion and preferable interaction between the cell membrane and the D-type amino acids ligands due to the homo-chirality preference of human body, also means better biocompatibility 186.

Correspondingly, easy cellular uptake of exogenetic materials can also cause higher biological effects. For example, Cui's group reported that D-GSH coated Au NCs can generate more ROS and exhibit higher PDT efficiency that L-GSH coated Au NCs in the treatment gastric carcinoma 99,187. Similarly, Xu's group also reported that D-Cys-MoO_2_ and D-Cys-MoO_2.83_ demonstrated higher PTT efficiency than the L-ones 6. For the cases in regulating the cell fate, mimicking nanozyme for gene editing, as well as in treating neurodegenerative diseases, majority of D-form NPs were reported to have better cellular internalization abilities, such as D-Cys-Cu_2-x_S NCs 78, D-GSH-UYTe 106, D-Cu_x_Co_y_S NPs 109, D-Fe_x_Cu_y_Se NPs 98, D-GSH-Au NPs 97,* etc*. However, Chen's group have reported a pair of L-/D-GSH-anchored selenium nanoparticles (G@Se NPs), and L-form exhibited stronger cell adhesion and uptake ability, as well as functioning in preventing oxidation damage in sulinoma cells. The detailed mechanism was summarized in relative section 4.

For higher chiral supraparticles, recent work from Liu's group also confirmed that the cellular uptake is also dependent on chiral morphology 186. They fabricated the Au nanooctopods possessing eight bent arms with a propeller-like structure. By studying the difference in cellular uptake efficiency of L-GSH, D-GSH, polyethylene glycol (PEG)-coated and racemic structures, they confirmed the that the chiral morphology can dominate the cell-selective uptake of chiral NPs (Figure 10B). This result also indicates that chiral morphology plays a more important role in cell interactions compared with chiral surface ligands. Due to our body's abundance of L-GSH, surface ligand exchange of chiral NPs by L-GSH may affect the chiral-ligand dependence of cellular uptake. In comparison, NPs with chiral morphologies could realize their functions without being affected by the chiral biomolecules numerous in living systems.

### Bio-safety evaluation

Biological nanomaterials used in biomedical field can be either bio-active or bio-inactive, but they must be easily accepted by organisms while meeting the requirements of functional use, and without causing side effects. Therefore, it is necessary to evaluate its biological toxicity and biocompatibility before use. After therapy, it is also necessary to evaluate the bio-safety. The scope of bio-safety assessment mainly includes *in vitro* and *in vivo* biocompatibilities.

#### *In vitro* biocompatibility

As addressed in the previous section of chiral-dependent cellular uptake, toxicity of NPs after entering the cell can be mainly determined by various factors, including particle size, chiral ligands, as well as the intrinsic toxicity of the inorganic cores. It is nothing that some chiral ligands can be used to reduce the intrinsic toxicity of the inorganic cores. For example, Chen group has reported a CdSe/CdS dot/rod nanostructure, that can be used for tumor PDT, the toxic results demonstrated that the CdS shells and the cysteine ligands used for fabricating the CdSe/CdS dots/rods are the main reasons for their low cytotoxicity 85.

In recent years, the toxicity of many kinds of chiral inorganic nanomaterials has been extensively studied. Overall, the toxic effect that determined by various factors of the NPs is dose-dependent, and highly associated with the final particle size, which mainly influence the physiological conditions of cell growth.

The intrinsic toxic mechanisms of nanoagents on cells can be summarized into the following categories: 1) Drugs interfere with the absorption, transportation and utilization of oxygen; 2) Inhibit enzyme system activity to cause cell damage; 3) Drugs destroy cell structure; 4) Drugs interfere with metabolic functions; 5) Drugs affect immunity features.

**Figure 11:**
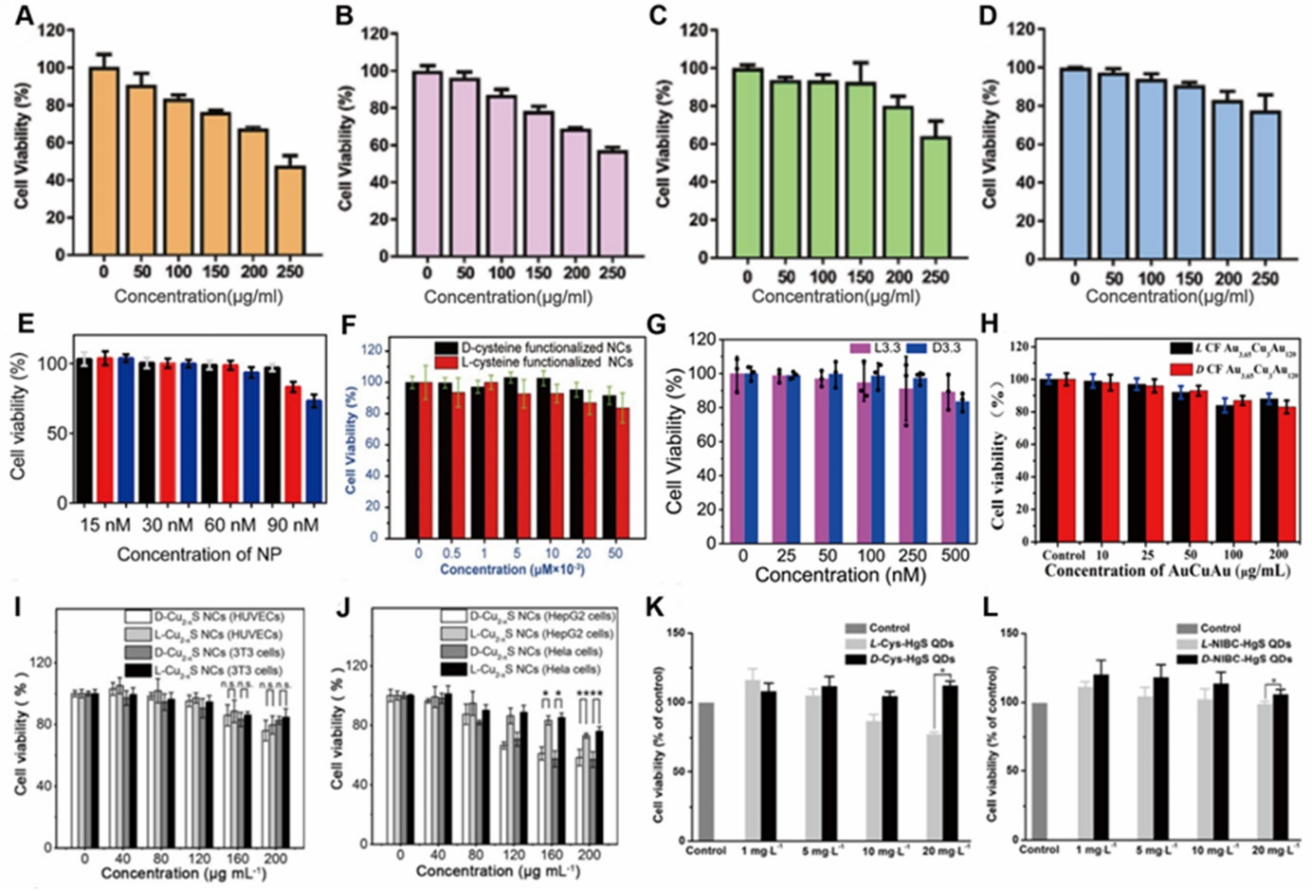
Summary of cyto-toxicity of recent chiral NPs. **A.** D-Cys-MoO_2_ NPs**. B.** L-Cys-MoO_2_ NPs; **C.** D-Cys-MoO_2.8_ NPs; **D.** L-Cys-MoO_2.8_ NPs; **E.** Cu_x_Co_y_S NPs; **F.** Cys-CdSe/CdS dot/rod NCs; **G.** L3.3 and D3.3; **H.** AuCuAu NRs;** I.** Cys-Cu_2-x_S NCs; **J.** Cys-Cu_2-x_S NCs;** K.** Cys-HgS QDs; **L.** NIBC-HgS QDs. **Panel A-D** are adapted with permission from 6, copyright 2020 Wiley-VCH. **Panel E** is adapted with permission from 109, copyright 2020 Wiley-VCH.** Panel F** is adapted with permission from 85, copyright 2019 the Royal Society of Chemistry. **Panel G** is adapted with permission from 97, copyright 2020 Springer Nature. **Panel H** is adapted with permission from 90, copyright 2020 Wiley-VCH. **Panel I-J** are adapted with permission from 78, copyright 2020 Wiley-VCH. **Panel K-L** are adapted with permission from 79, copyright 2019 Elsevier.

It has been found that chiral inorganic nanomaterials have biocompatibility in a variety of cell lines, such as HeLa, IMR-90 cells, SH-SY5Y, INS-1 cells lines, HUVECs, 3T3 cells, HepG2 cells, *etc*. For example, transition metal chiral nanomaterials induced by chiral amino acids have biocompatibility within a certain concentration range. The chiral molybdenum oxide studied by Xu *et al.* 6 is well tolerated in HeLa cells (Figure 11A-D). The tolerance of cells to different valence states of molybdenum oxide was different, as they adopted different particle diameters. The results showed that the biocompatibility of MoO_2.8_ was better than that of MoO_2_, which can be reflected by the cell viability assays. By which, 150 μg/mL MoO_2.8_ or 50 μg/mL MoO_2_ can be used for keeping more than 85% cells alive. Bio-TEM was also employed to evaluate the damages to the ultra-structures of the cells. At the safe concentration, MoO_3-x_ was shown that can be successfully internalized to the HeLa cells. The membrane structure was integrity and the cytoplasm was uniform without vacuolation, which also confirmed their biocompatibility were suitable for biomedical prospects.

Similarly, Kuang *et al.* 109 has shown that chiral Pen-Cu_x_Co_y_S can achieve a cell survival rate of up to 95% at a concentration of 60 nM (Figure 11E). Chen *et al.*'s 85 D-/L-Cys Coated CdSe/CdS also showed better biocompatibility at higher concentrations (Figure 11F). Cu_2-x_S 78 exhibits different cytotoxicity in different cells. The toxicity in normal cells (HUVECs and 3T3 cells) is much lower than that in tumor cells (HepG2 and HeLa cells) (Figure 11I-J). In addition, the toxicity of materials induced by different ligands to the same cell is also inconsistent. For example, NIBC-HgS 79 has much better biocompatibility than Cys-HgS QDs (Figure 11K-L). Similarly, studies on the biocompatibility of noble metals have also shown that cells are very optimistic about their tolerance. For example, when the concentration of chiral gold nanomaterials studied by Tang *et al.* 97 is 250 nM, the survival rate of human neuroblastoma cell line SH-SY5Y is over 85% (Figure 11G). The Cys-Phe-modified chiral AuCuAu HNRs 90 studied by Xu *et al.* also have good biocompatibility (Figure 11H). Cytotoxicity of other recent transition metal chiral nanomaterials to various cell types were summarized in Table 3.

#### *In vivo* compatibility

##### Main organs damages

For biomedical applications, nanomaterials were generally delivered into model animals by both intravenous and *in situ* injections. Either way, the nano-agents could participate in the blood circulation and enter main organs in animals. Generally, immunohistochemical methods, such as hematoxylin-eosin (HE), were employed for evaluation the histocompatibility of chiral nanomaterials *in vivo*.

**Figure 12:**
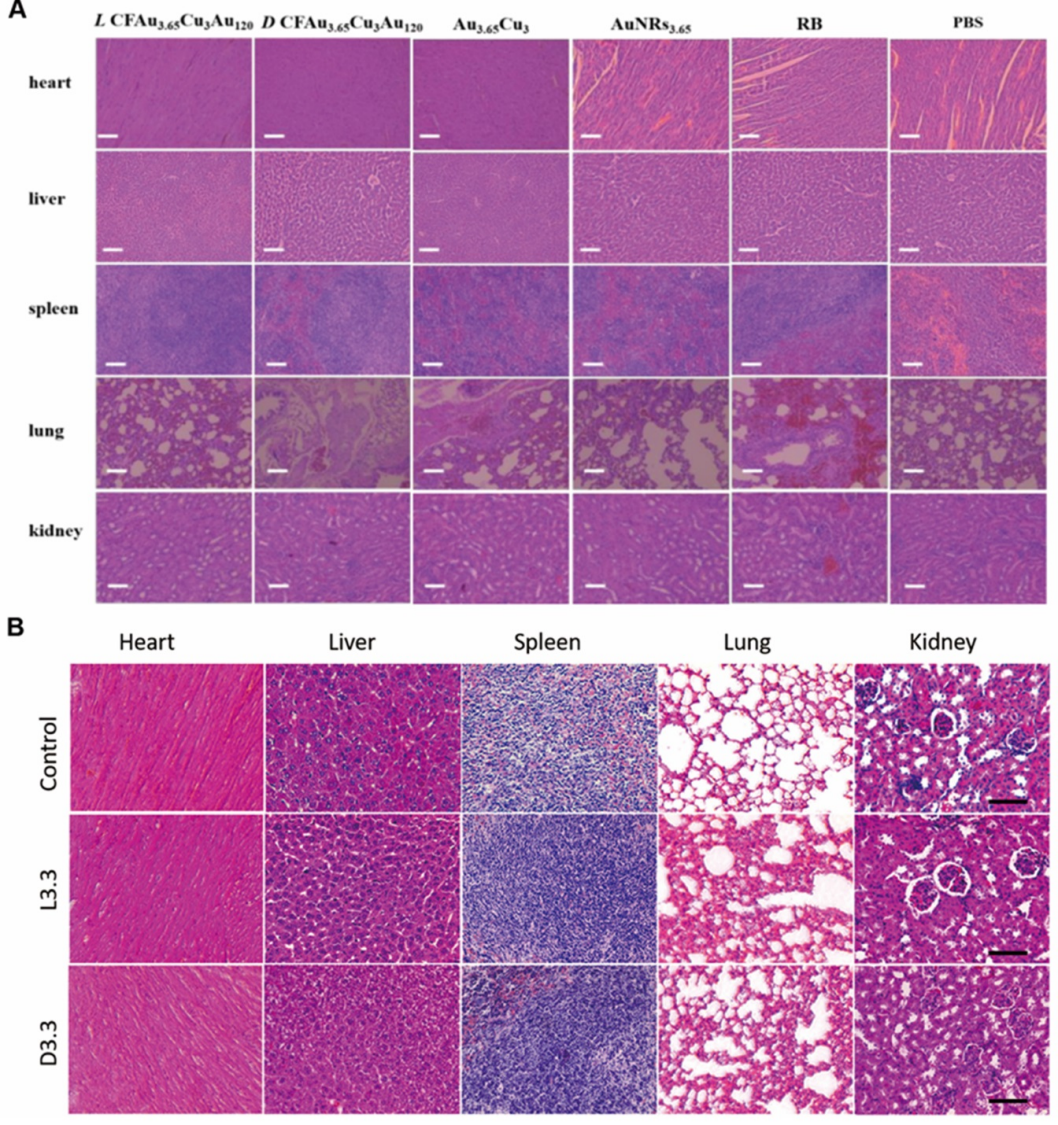
Main organ damages evaluation caused by different chiral NPs after intravenous and *in situ* injection into mice. **A.** AuCuAu NRs; **B.** L3.3 and D3.3. **Panel A:** Adapted with permission from 90, copyright 2020 Wiley-VCH. **Panel B:** Adapted with permission from 97, copyright 2020 Springer Nature.

Xu* et al.* 90 detected the toxicity of chiral AuCuAu HNRs, they injected 50 µg/mL L-CF-Au_3.65_Cu_3_Au_120_ HNRs into each mouse through the tail vein at 0, 5, 10, 15 and 20 d, then collected the heart, liver, spleen, lung and kidney of each mouse. Immunohistochemical results and analysis showed that there was no obvious toxicity in main metabolism organs, including liver and kidney (Figure 12A).

Tang *et al.* 97 researched L3.3 or D3.3 gold nanoparticles. 48 h after injection of L3.3 or D3.3 also through tail vein, HE stained tissue sections of the heart, liver, spleen, lung and kidney showed also no obvious organ damage or inflammation either. This shows that it has excellent *in vivo* biocompatibility (Figure 12B).

For nanomaterials used for phototherapy through *in situ* tumor injection, such as L-/D-Cys-MoO_3-x_ 80, main organs damages were also evaluated by immunohistochemical methods. HE staining results have also shown there was no organ damages. In addition, combined with immunofluorescence methods, tumor cell apoptosis was also detected.

##### Biodistribution and Clearance

Intravenously administered nanoparticles are cleared from the body through two main pathways: hepatobiliary elimination and renal elimination 188.

**Figure 13:**
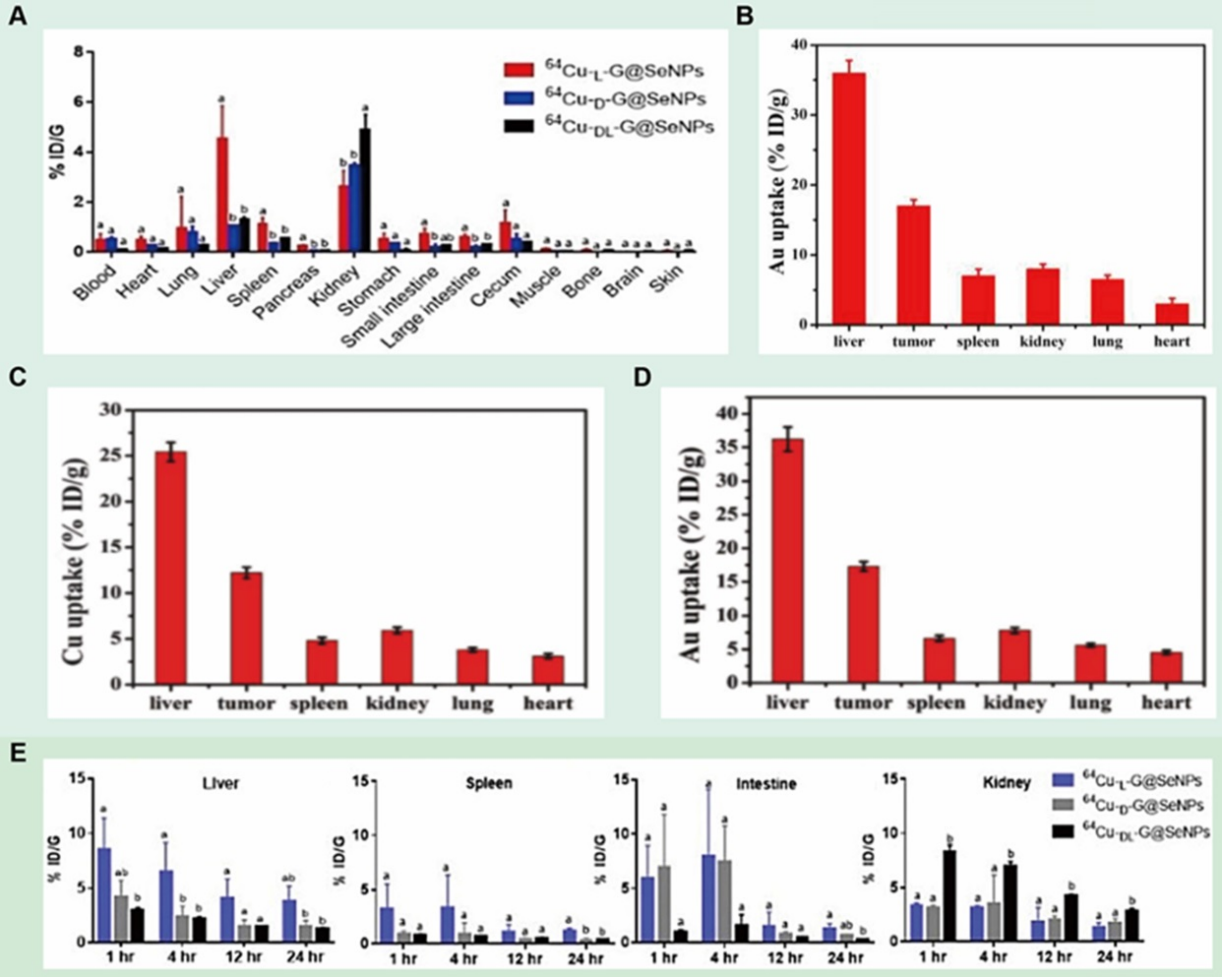
Biodistribution and clearance of different chiral NPs after intravenous injection into mice.** A.** GSH@Se NPs; **B.** SS15; **C.** AuCuAu NRs; **D.** AuCuAu NRs; **E.** GSH@Se NPs. **Panel A** is adapted with permission from 110, copyright 2019 Wiley-VCH. **Panel B** is adapted with permission from 89, copyright 2017 Wiley-VCH. **Panel C-D** are adapted with permission from 90, copyright 2020 Wiley-VCH. **Panel E** is adapted with permission from 110, copyright 2019 Wiley-VCH.

GSH@Se NPs 110 of different chirality were injected intravenously. The results of the study showed that most of the NPs of different chirality accumulated in the liver and kidney, and a small amount were also accumulated in other organs such as the heart, lungs, spleen, and intestines (Figure 13A). PET imaging confirmed that Cu was reduced, quickly transported to the liver and then eliminated through the bladder (Figure 13E).

Regarding the distribution of nanomaterials in mice affected by tumors, Xu* et al.* 89 studied SS15-D-Cys-Au NPs. The results of the *in vivo* test showed that the two strong signals were clearly distinguishable at the tumor site 24 h after injection compared with 0 h, which indicated that SS15-D-Cys-Au NPs may become an imaging agent for CT imaging and PA imaging. The distribution of the gold element in the tissue is determined by inductively coupled plasma mass spectrometry (ICP-MS), which shows the efficient accumulation of the developed nano-components. It can be clearly observed that the accumulation in the liver is the most, followed by the accumulation in the tumor (Figure 13B).

Similarly, the composition of Au and Cu in the heart, liver, spleen, lung, kidney and tumor sites of chiral AuCuAu HNRs 90 was determined by ICP-MS. The results are consistent with the above materials. In general, these particles mainly exist in organs such as liver and kidney. It shows good excretion of NPs, which is probably due to the accumulation of redox-mediated biodegradable products in tumors (Figure 13C-D).

## Conclusion and Perspective

Chirality is one of the basic characteristics of molecular systems which are ubiquitous in nature, and plays an important role in biological administrations 189,190. However, most of chiral materials that have been studied are intrinsically chiral systems such as chiral molecules and biomolecules or inorganic salts at the molecular level. Until recent, chirality has been introduced into inorganic materials at nanoscale with great progresses in chemical synthesis and multidisciplinary bio-applications. Nonetheless, it is worth noting that the research on chiral inorganic nanomaterials for biological issues is still in its infancy. Most chiral nanomaterials are still in the stage of animal experiments or even theoretical levels. Substantial attentions should be paid for more advanced chiral synthesis and design, more detailed understandings on chiral matter-biointerface interactions, and more diversed front-line bioapplications to broaden the scope of chiral nanotechnologies in biomedicine and bioengineering. In particular, a perspective view about the future development on stereo-synthesis, biocompatibility evaluations, and emerging bioapplications based on stereospecific chiral nanosystems is outlined as below:

### Advanced stereo-synthesis

Although many chiral inorganic nanostructures have been reported in previous sections, in-depth discovery of chiral structures through advanced techniques with enhanced chirality or higher dimensional chirality is to some extent increasingly imperative or even attractive. For example, biomimetic time-dependent evolutions of chiral plasmonic particles *via* dynamic control over the chemical environment during the synthesis could be applied for manipulating the temporal development of chirality which rises the concept of 4D plasmonic chirality for modulating the synthesis of chiral nanostructures 191. Further, artificial intelligence (AI) enables *in situ* deep learning for real-time management of chiral synthesis which has recently been applied for fabrication of intrinsic chiral CsPbX_3_ (X=Cl, Br, I) perovskites whose chiral feature (lattice dislocations or defects) are often hard to be observed and separated from their enantiomers *via* traditional techniques 192. In a meanwhile, other principles such as chiral self-sorting 193,194, majority rules 195, and chiral environment or stimuli 4,5,49,196 are generally employed for attaining precisely controlled chiral nano-objects which envisions next revolution on the stereo-synthesis of chiral nanomaterials. Last, for the development of biomedicines such as artificial enzymes, biosensors or nanoplatforms for neurological diseases, biochemical stability of chiral nanostructures in cellular microenvironments would be another critical aspect that one should always keep in mind. The biomimetic helicoid NPs, for instance, have achieved recently with high *g*-values, however their penetration capabilities, long-term stability and biodegradation possibilities/routs are still under debate which may be improved in future by surface modification strategies such as interface engineering, encapsulations, biomineralizations, *etc.*

### Biocompatibility evaluations

Despite the rapid development of nanotechnology, the clinical translation still has faced significant hurdles. Biocompatibility, including toxicity, biodegradability, distribution inside the organism, immunogenicity, and clearance from the body, is the main concern before clinical trials. Although various methods, such as surface modification and chiral induction, have showed the advantages in reducing their toxicity, explicitation of the detailed biological effects, including different levels on gene-protein-metabolite during therapy and diagnosis is still a challenge. With the development of the biological high-throughput technologies, various biological mechanisms of different drugs have been interpreted. However, the applications of the high-throughput technologies such as proteomics, transcriptomics, metabonomics, as well as bioinformatics to nanomedicine is still very scarce. Integration and combination these technologies to nanomedicine can contribute to accelerate the shortenings of the gap to clinical transformations, and also promote the research of nanomedicine to a deeper and higher level.

### Emerging bio-applications

As summarized in previous sections, chiral inorganic nanomaterials have been extensively applied for synergistic diagnosis and treatment spanning from biosensing and bioimaging, to regulation of cellular activity, phototherapies of cancer, and neurodegenerative diseases treatment. Nonetheless, in light by the unique physical and chemical properties of chiral inorganic nanomaterials, one can clearly assert there should be no boundary for pursing chirality-dependent bio-applications due to the chiral nature of nature. CPL-active semiconductor nanocrystals, for instance, are widely considered as both ideal high-resolution markers for bioimaging, and highly sensitive photosensitizers for theranostics. X Chen and X Zhao for example 88, reported recently that alkynyl-protected Au_10_ chiral clusters could exhibit enhanced chirality-dependent radiotherapy when irradiated by X-ray. However, current CPL-active chiral nanostructures only exhibit enantioselective CPL signals in visible range (<800 nm) probably because of the detection/excitation limits of CPL spectrometer (CPL-300 from Jasco). For this matter, applications of CPL properties on biological issues are seriously limited by low tissue penetration depth and phototoxicity to healthy tissues. It is then promising that with development of CPL testing techniques to NIR region or new species of CPL-active nanomaterials with NIR emissions or upconverted properties 197, lanthanide UCNPs for instance, there would be plenty of room available for more CPL-based bioinspired applications in future.

### Main concerns to clinical translations

In addition to the perspectives discussed in the previous section, there are still two main challenges to be solved before actual clinical translations.

(i) The long-term toxicity *in vivo* is a major concern, which has also attracted much attentions for a long time. Through chiral-ligands were demonstrated that can help to enhance the biocompatibility of the NPs and facilitate the clearance from the body through the excretion organs, however, due to the extra small size of the NPs and the complexity of the body physiological environment, the remained NPs have the possibility to return to the blood, participate in longer circulation, and further accumulate in different organs and cause some systemic damage, such as thrombus, endothelial leakiness, destroy the tight junctions (TJ) of the blood brain barrier (BBB). Hence, exploring in-depth at multiple biological levels of the *in vivo* long-term toxicity is the premise before clinical transformation.

(ii) In this work, we have reviewed a great deal of chiral NPs which have the biomedical translation prospects. Many of them require CPL excitation to realize their function. Most materials are excited by NIR light, and some of them were reported can be excited by visible light. However, due to the limited depth of laser penetration through human tissue, the current strategies is difficult to achieve effective treatment of target-sites located deep in the tissues, especially for tumors. Therefore, to further promote the clinical transformation of chiral NPs used for phototherapy, especially for tumors located deep in the tissue, it is very necessary to develop more chiral NPs which can be excited by NIR Ⅱ lasers to improve the tissue penetration depth, and facilitates the treatments.

In sum, although different types of chiral nanomaterials have their own pros and cons, the clinical applications of chiral nanomaterials are usually subject to unknown targeting effects, the clearance of nanomedicines, long-term biological toxicity, and tissue penetration. Therefore, the development of new chiral nanomaterials with high efficiency, stability and safety will become a top priority for next generation of nanomedicines.

## Figures and Tables

**Table 1:** Summary of biochemical and pharmaceutical detections based on chiral semiconductor-NPs

Chiral nanostructures	Type of chirality	Analyte	Working concentration/Limit of detection (LOD)		Applications	Ref
**Cd**						
L-/D-Cys-CdS QDs	LIC	Ni^2+^	10-60 µМ/7.33 µМ		Detection	117
		Co^2+^	4-80 µМ/1.13 µМ			
		L-Morphine D-Methamphetamine	-/-			118
CdTe QDs	LIC	D-Cys	Enantiomeric excess of D-Cys: -100%-100%/-		Recognition	119
N-Acetyl-L-Cysteine (NALC)-CdSe/CdS QDs	LIC	Tyr enantiomers	4.0×10^-5^-1.2×10^-3^ M/-		Recognition	120
Au NRs and CdTe@CdS QDs core-satellite structure	CAM	Cys enantiomers	-/10%		Recognition and quantification	121
L-Pen-Cys-CdS QDs	LIC	Cd^2+^	65-200 µM/59.7 µM		Detection	122
		S^2-^	7-125 µM/1.6 µM			
[Cys-CdS QDs]-Fe^3+^	LIC	Pyrophosphate (PPi)	0.5-10 µM/0.11 µM		Detection	123
DPA/Cys-CdS QDs	LIC	Cu^2+^	0.50-2.25 µM/0.34 µM		Detection	124
		Glucose	50-250 µM/31 µM			115
NALC-CdTe QDs	LIC	Phenylglycinol (PG) enantiomers	10^-7-^10^-5^ M/10^-7^ M		Recognition	125
L-/D-Cys-CdSe QDs	LIC	Pb^2+^	-/4.9 nM		Detection	126
L-/D-Cys-CdTe NPs	LIC	Recognize the specific sequence: GAT′ ATC	-/-		Recognition (and then cleave the DNA) (Site-selective photoinduced DNA cleavage and profiling)	10
β- Cyclodextrin-CdSe/ZnS QDs	LIC	Pen enantiomers	L-Pen 0.8-5.0 mg/L/0.2 mg/L	D-Pen 0.1-5.0 mg/L/0.06 mg/L	Recognition	127
β-Cyclodextrin-functionalized CdSe/ZnS QDs with receptor-bound Rhodamine B	LIC	Aromatic amino acids enantiomers (Phe and Tyr)	-/1.0×10^-6^ M (4-hydroxytoluene,1-adamantanecarboxylic acid and 4-nitrophenol)		Detection	128
α/β-Cyclodextrin-CdSe/ZnS QDs	LIC	Met enantiomers Tyr enantiomers	5×10^-8^-5×10^-5^ M/-		Recognition	129
Methyl ester NALC capped-CdSe/ZnS QDs	LIC	R/S-Ketoprofen R/S-Naproxen R/S-Flurbiprofen	-/-		Detection	130
L-/D-Cys-CdSe/ZnS QDs	LIC	Carnitine enantiomers	0.05-0.3 mM/ 0.013 mM (without D-Carnitine) 0.016 mM (without L-Carnitine)		Recognition and quantification	131
ZnTPyP-CdTe QDs	CAM	D-Pro	1.0×10^-9^-1.5×10^-7^ M/4.46×10^-10^ M		Detection	47
		D-Lys	1.0×10^-9^-1.5×10^-8^ M/7.13×10^-11^ M			
		L-Ser	1.0×10-9-5.0×10^-9^ M/3.35×10^-11^ M			
**Zn**						
β-Cyclodextrin-Mn-ZnS QDs	LIC	L-/D-Trp	5.4 nM-6.0 µM/5.4 nM		Detection	132
ZnO@Cys NPs	LIC	Dopamine (DA) content	26.3-68.5 μM/0.791 μM		Detection	133
**Cu**						
L-/D-Pen-Fe_x_Cu_y_Se NPs	LIC	Recognition pentapeptide on Aβ42 fibrils	-/-		Recognition	98
**Mo**						
Magnetic nanoparticles (MNPs)-MoS_2_ QDs	LIC	Dual-mode detection	PL detection	CD detection	Detection	134
		Avian influenza A: H4N6	128-0.0012 HAU/50 µL/0.00403 HAU/50 µL	128-0.0128 HAU/50 µL/0.0381 HAU/50 µL		
		H5N1	10 pg/mL-10 µg/mL/7.35 pg/mL	100 pg/mL-10 µg/mL/80.92 pg/mL		
Cys-MoO_2_/GO_x_	LIC	D-Glucose	200-1000 μM/0.446 μM		Recognition	135
MoO_2_ NPs	LIC	Hg^2+^	0.1-4 nM/0.08 nM (D-Cys-MnO_2_ NPs)	0.1-4 nM/0.12 nM (L-Cys-MnO_2_ NPs)	Detection	136

*CAM represents for Chiral-Assemblies and Morphologies, while LIC represents for Ligand Induced Chirality.

**Table 2:** Summary of biochemical and pharmaceutical detections based on chiral noble-NPs

Chiral nanostructures	Type of chirality	Analyte	Working concentration/LOD		Applications	Reference
**Au**						
Au NRs	CAM	Carnitine enantiomers	Enantiometric excess of L-Carnitine: -100%-100%		Recognition	142
		Gln enantiomers	D-Gln: -100%-100%		Recognition	143
		DNA	-/3.7 aM		Detection	144
		L-/D-Cys	L-Cys: -/1.40 µM D-Cys: -/0.80 µM		Detection	145
		L-/D-GSH	L-GSH: -/1.93 µM			
L-Cys-Au NRs	LIC	Cu^2 +^	20 pM-5 nM/7 pM		Detection	146
Au nanosphere clusters	LIC	L-/D-Cys	9×10^-6^ M/9×10^-7^ M		Detection	147
Pen-Au NPs	LIC	3,4-dihy-droxyphenylalanine (DOPA) enantiomers	-/-		Recognition	148
Au NRs	CAM	Cys enantiomers	L-Cys: 0.625-2.50 µM/0.325 µM		Detection	149
Au NP oligomers	CAM	Antibiotics	0.05-5 ng/mL/0.014 ng/mL		Detection	150
Au NP dimers	CAM	Bisphenol A (BPA)	0.02-5 ng/mL/0.008 ng/mL		Detection	151
		Ag+	0.005-10 nM/2 pM		Detection	152
		DNA methyltransferase	M.SssI MTase: 0.5-150 U/mL/0.27 U/mL		Detection	153
		8-hydroxy-2′-deoxyguanosine (8-OHdG)	0.05-2 nM/33 pM		Detection	154
		Intracellular ATP	1.5-4.2 mM/0.2 mM		Detection	155
		Intracellular telomerase	0.8×10^-12^-32×10^-12^ IU/1.7×10^-15^ IU		Detection	155
		L-Cys	0.05-5 nM/20 pM		Recognition and quantification	156
		Alpha-fetoprotein (AFP)	0.02-5 ng/mL/11 pg/mL		Detection	157
Anti-BPA antibody and coating antigen coated Au20 and Au10 dimers	CAM	BPA	0.05-10 ng/mL/0.02 ng/mL		Detection	158
Au NR-UCNP tetramers	CAM	DNA	3.3×10^-8^-3.3×10^-5^ nM/13.2 aM		Detection	159
Au shell core-Au NP satellite	CAM	Ochratoxin A (OTA)	0.1-5 pg/mL/0.037 pg/mL		Detection	160
Core-satellite Au NP networks	CAM	L-/D-Carnitine	-/At pM level		Recognition	161
L-/D-Cys modified Au electrode	LIC	L-/D-Carnitine	10^-6^-10^-2^ M/-		Recognition	162
Au films	CAM	Probe the second structure of Concanavalin A with a high β-sheet content	-/-		Recognition	46
Au NP chiral pyramids	CAM	DNA	10-5000 aM/3.4 aM		Detection	163
Au NP dimer with metal deposition	CAM	DNA	160 zM-1.6 pM/17 zM		Detection	164
Au NR side by side structure	CAM	Hg^2+^	0.05-10 ng/mL/0.03 ng/mL		Detection	165
Au-UCNP pyramids	CAM	Intracellular miRNA	0.073-43.65 fmol/10 μg (RNA)/0.03 fmol/10 μg (RNA)		Detection and quantification	166
Au NRs	CAM	Amyloid fibrils	-/-		Detection	113
Two-layer core-satellite Au nanostructures (C_30_S_5_S_10_ NS)	CAM	Intracellular miRNA (Dual-signal method)	CD detection 0.011-20.94 amol/ng (RNA)/0.0051 amol/ng (RNA)	Raman signal 0.052-34.98 amol/ng (RNA)/2.81×10^-2^ amol/ng (RNA)	Detection and quantification	140
L-/D-GSH-Au NPs (3.3 nm)	LIC	Aβ peptides	-/-		Recognition	97
Au NPs	CAM	*Opisthorchis viverrini* (Ov) antigen in urine	-/23.4 ng/mL		Detection	167
GO-Au NP structures	CAM	MiR-21	0.07-13.68 amol/ng (RNA)/0.03 amol/ng (RNA)		Detection	139
		EpCAM	8.47-74.78 pg/mL/3.63 pg/mL			
L-tartaric acid-capped Au NPs	LIC	L-/D-Mandelic acid (MA)	Enantiometric excess of L-MA: -100%-100%		Recognition	168
Au@AgAu yolk-shell NRs-Au NPs core-satellite structure	CAM	Zn^2+^	-/38.7 × 10^-6^ M (per 10^6^ cells)		Detection	169
Au@Ag-NR dimers	CAM	PSA	0.1-50 aM/0.076 aM		Detection	137
Chiral Ag NP-Au NP dimers	CAM	Environmental pollutants	Microcystin-LR (MCLR): 0.002-0.5 ng/mL/8×10^-13^ M		Detection	170
		PSA	1×10^-9^-1×10^-6^ ng/mL/1.5 ×10^-20^ M			
Ag@Au core-shell NP (CSNP) assemblies	CAM	Circulating tumor cells with HER2 overexpression	In SK-BR-3 cells: 50-10^5^ cells/mL/10±6 cells/mL		Separation	138
Au@Ag core-shell NPs	CAM	OTA	1-50 pg/mL/0.16 pg/mL		Detection	171
Chiral Cys modified individual Au@Ag core-shell nanocuboids (CSNCs)	LIC	Extended helical conformation of chemisorbed cysteine molecules	-/-		Recognition	172
NALC-Au NPs	LIC	L-Tyr	-/-		Detection and separation	173
D-/L-Cys-Au NPs	LIC	Racemic propylene oxide (PO)	0.5 M-/-		Separation	174
Bovine serum albumin enantioselective films coupled with Ag-enhanced Au NPs	LIC	Quantitatively analysis of chiral amino acids	L-Trp: 1.33×10^-12^-1×10^-9^ M/5×10^-13^ M		Detection	175
L-/D-Au gammadion structures	IC	Sensing proteins with β-sheet contents	At pg level/At pg level		Detection	176
**Ag**						
Uridine 5′-triphosphate (UTP)-capped Ag NP	LIC	Racemic Cys	D-Cys: 0.1-100 μM/100 nM (limit of separation) L-Cys: -/100 nM (limit of separation)		Separation and detection	177
L-Arg-Zn Tetraphenylporphyrin-Ag NPs (L-Arg-ZnTPPS-Ag NPs)	LIC	L-His	5.0×10^-6^-2.5×10^-5^ M/-		Recognition	178
DNAzyme-induced asymmetric Ag NP dimers	LIC	Pb^2+^	0.05-10 ng/mL/0.02 ng/mL		Detection	179
L-/D-Au NPs	CAM	Enantiomers of cysteine containing disulfide bond	-/-		Recognition	180
Chiral nanostructured Au films (CNAFs)	CAM	Various enantiomers	-/-		Recognition	181
**Pt**						
L-Trp-rGO@ Pt NPs/ glassy carbon electrode (L-Trp-rGO@Pt NPs/GCE) GCE	LIC	DOPA enantiomers	5.0×10^-8^-5.0×10^-3^ M/1.7×10^-8^ M		Detection	182
**Semiconductor and noble metal composite**						
A superstructure of chiral gold nanohybrids and QDs	CAM	H5N1	10 pg-10 μg/mL/1 pg/mL		Detection	183
		H4N6	In PBS buffer 100-0.01 HAU/50 μL/0.0268 HAU/50 μL	In complex 100-0.01 HAU/50 μL/0.0315 HAU/50 μL		
		Infectious bronchitis virus (IBV)	10^2^-10^4^ EID/50 μL /47.91 EID/50 μL			
		Fowl adenoviruses-9 (FAdVs-9)	-/33.64 PFU/mL			

*CAM represents for Chiral-Assemblies and Morphologies, LIC represents for Ligand Induced Chirality while IC represents for Intrinsic Chirality.

**Table 3:** Cytotoxicity of recent transition metal chiral nanomaterials

Material	Cell type	LTC	Reference
D-/L-Cys-MoO_2.8_	HeLa	200 µg/mL	6
D-/L-Cys-MoO_2_	HeLa	50 µg/mL	6
D-/L-Cys-Phe-AuCuAu HNRs	HeLa	50 µg/mL	90
D-/L-Cys-CdSe/ZnS QDs	EAC	20 µM	114
Pen-Cu_x_Co_y_S NPs	IMR-90	60 nM	109
D-/L-GSH-Au NPs	SH-SY5Y	250 nM	97
D-/L-NIBC-HgS QDs	INS-1	20 mg/mL	79
D-/L-Cys-HgS QDs	INS-1	10 mg/mL	79
D-/L-Cys-CdSe/CdS dot/rod NCs	HeLa	5 nΜ	85
D-/L-Cys-Cu_2-x_S NCs	HUVECs	120 µg/mL	78
D-/L-Cys-Cu_2-x_S NCs	3T3	120 µg/mL	78
L-Cys-Cu_2-x_S NCs	HepG2	120 µg/mL	78
D-Cys-Cu_2-x_S NCs	HepG2	80 µg/mL	78
L-Cys-Cu_2-x_S NCs	HeLa	120 µg/mL	78
D-Cys-Cu_2-x_S NCs	HeLa	80 µg/mL	78

## References

[1] Lebreton G, Géminard C, Lapraz F, Pyrpassopoulos S, Cerezo D, Spéder P (2018). Molecular to organismal chirality is induced by the conserved myosin 1D. Science.

[2] Ma W, Hao C, Sun M, Xu L, Xu C, Kuang H (2018). Tuning of chiral construction, structural diversity, scale transformation and chiroptical applications. Mater Horiz.

[3] Salem DP, Landry MP, Bisker G, Ahn J, Kruss S, Strano MS (2016). Chirality dependent corona phase molecular recognition of DNA-wrapped carbon nanotubes. Carbon.

[4] Yeom J, Yeom B, Chan H, Smith KW, Dominguez-Medina S, Bahng JH (2015). Chiral templating of self-assembling nanostructures by circularly polarized light. Nat Mater.

[5] Kim JY, Yeom J, Zhao G, Calcaterra H, Munn J, Zhang P (2019). Assembly of gold nanoparticles into chiral superstructures driven by circularly polarized light. J Am Chem Soc.

[6] Li Y, Miao Z, Shang Z, Cai Y, Cheng J, Xu X (2019). A visible- and NIR-light responsive photothermal therapy agent by chirality-dependent MoO_3-x_ nanoparticles. Adv Funct Mater.

[7] Li Y, Cheng J, Li J, Zhu X, He T, Chen R (2018). Tunable chiroptical properties from the plasmonic band to metal-ligand charge transfer band of cysteine-capped molybdenum oxide nanoparticles. Angew Chem Int Ed Engl.

[8] Das RK, Zouani OF, Labrugère C, Oda R, Durrieu MC (2013). Influence of nanohelical shape and periodicity on stem cell fate. ACS Nano.

[9] Qu A, Sun M, Kim JY, Xu L, Hao C, Ma W (2021). Stimulation of neural stem cell differentiation by circularly polarized light transduced by chiral nanoassemblies. Nat Biomed Eng.

[10] Sun M, Xu L, Qu A, Zhao P, Hao T, Ma W (2018). Site-selective photoinduced cleavage and profiling of DNA by chiral semiconductor nanoparticles. Nat Chem.

[11] Zhao X, Zang SQ, Chen X (2020). Stereospecific interactions between chiral inorganic nanomaterials and biological systems. Chem Soc Rev.

[12] Ma W, Xu L, de Moura AF, Wu X, Kuang H, Xu C (2017). Chiral inorganic nanostructures. Chem Rev.

[13] Ben-Moshe A, Maoz BM, Govorov AO, Markovich G (2013). Chirality and chiroptical effects in inorganic nanocrystal systems with plasmon and exciton resonances. Chem Soc Rev.

[14] Li Y, Wang X, Miao J, Li J, Zhu X, Chen R (2020). Chiral transition metal oxides: Synthesis, chiral origins, and perspectives. Adv Mater.

[15] Dong Y, Zhang Y, Li X, Feng Y, Zhang H, Xu J (2019). Chiral perovskites: Promising materials toward next-generation optoelectronics. Small.

[16] Long G, Sabatini R, Saidaminov MI, Lakhwani G, Rasmita A, Liu X (2020). Chiral-perovskite optoelectronics. Nat Rev Mater.

[17] Han Z, Wang K, Guo Y, Chen W, Zhang J, Zhang X (2019). Cation-induced chirality in a bifunctional metal-organic framework for quantitative enantioselective recognition. Nat Commun.

[18] Zhao T, Han J, Jin X, Zhou M, Liu Y, Duan P (2020). Dual-mode induction of tunable circularly polarized luminescence from chiral metal-organic frameworks. Research.

[19] Shen B, Kim Y, Lee M (2020). Supramolecular chiral 2D materials and emerging functions. Adv Mater.

[20] Solomon ML, Saleh AAE, Poulikakos LV, Abendroth JM, Tadesse LF, Dionne JA (2020). Nanophotonic platforms for chiral sensing and separation. Acc Chem Res.

[21] Fan J, Kotov NA (2020). Chiral nanoceramics. Adv Mater.

[22] Cheng J, Hill EH, Zheng Y, He T, Liu Y (2018). Optically active plasmonic resonance in self-assembled nanostructures. Mater Chem Front.

[23] Mcfadden CF, Cremer PS, Gellman AJ (1996). Adsorption of chiral alcohols on “chiral” metal surfaces. Langmuir.

[24] Dolamic I, Knoppe S, Dass A, Bürgi T (2012). First enantioseparation and circular dichroism spectra of Au38 clusters protected by achiral ligands. Nat Commun.

[25] Kuno J, Miyake K, Katao S, Kawai T, Nakashima T (2020). Enhanced enantioselectivity in the synthesis of mercury sulfide nanoparticles through ostwald ripening. Chem Mater.

[26] Hananel U, Ben-Moshe A, Tal D, Markovich G (2019). Enantiomeric control of intrinsically chiral nanocrystals. Adv Mater.

[27] Mukhina MV, Maslov VG, Baranov AV, Fedorov AV, Orlova AO, Purcell-Milton F (2015). Intrinsic chirality of CdSe/ZnS quantum dots and quantum rods. Nano Lett.

[28] Zhuang TT, Li Y, Gao X, Wei M, García de Arquer FP, Todorović P (2020). Regioselective magnetization in semiconducting nanorods. Nat Nanotechnol.

[29] Bürgi CGT (2006). Chiral N-isobutyryl-cysteine protected gold nanoparticles: Preparation, size selection, and optical activity in the UV-vis and infrared. J Am Chem Soc.

[30] Huang P, Pandoli O, Wang X, Wang Z, Li Z, Zhang C (2012). Chiral guanosine 5′-monophosphate-capped gold nanoflowers: Controllable synthesis, characterization, surface-enhanced raman scattering activity, cellular imaging and photothermal therapy. Nano Res.

[31] Jiang S, Chekini M, Qu ZB, Wang Y, Yeltik A, Liu Y (2017). Chiral ceramic nanoparticles and peptide catalysis. J Am Chem Soc.

[32] Tohgha U, Deol KK, Porter AG, Bartko SG, Choi JK, Leonard BM (2013). Ligand induced circular dichroism and circularly polarized luminescence in CdSe quantum dots. ACS Nano.

[33] Gao X, Zhang X, Zhao L, Huang P, Han B, Lv J (2018). Distinct excitonic circular dichroism between wurtzite and zincblende CdSe nanoplatelets. Nano Lett.

[34] Kuzyk A, Schreiber R, Fan Z, Pardatscher G, Roller EM, Högele A (2012). DNA-based self-assembly of chiral plasmonic nanostructures with tailored optical response. Nature.

[35] Chen CL, Rosi NL (2010). Peptide-based methods for the preparation of nanostructured inorganic materials. Angew Chem Int Ed Engl.

[36] Mokashi-Punekar S, Zhou Y, Brooks SC, Rosi NL (2020). Construction of chiral, helical nanoparticle superstructures: Progress and prospects. Adv Mater.

[37] Merg AD, Boatz JC, Mandal A, Zhao G, Mokashi-Punekar S, Liu C (2016). Peptide-directed assembly of single-helical gold nanoparticle superstructures exhibiting intense chiroptical activity. J Am Chem Soc.

[38] Bagiński M, Tupikowska M, González-Rubio G, Wójcik M, Lewandowski W (2019). Shaping liquid crystals with gold nanoparticles: Helical assemblies with tunable and hierarchical structures *via* thin-film cooperative interactions. Adv Mater.

[39] Wang ZY, Zhang NN, Li JC, Lu J, Zhao L, Fang XD (2021). Serum albumin guided plasmonic nanoassemblies with opposite chiralities. Soft Matter.

[40] Lu J, Xue Y, Bernardino K, Zhang NN, Gomes WR, Ramesar NS (2021). Enhanced optical asymmetry in supramolecular chiroplasmonic assemblies with long-range order. Science.

[41] Yan W, Xu L, Xu C, Ma W, Kuang H, Wang L (2012). Self-assembly of chiral nanoparticle pyramids with strong R/S optical activity. J Am Chem Soc.

[42] Schlesinger M, Giese M, Blusch LK, Hamad WY, MacLachlan MJ (2015). Chiral nematic cellulose-gold nanoparticle composites from mesoporous photonic cellulose. Chem Comm.

[43] Chu G, Wang X, Chen T, Gao J, Gai F, Wang Y (2015). Optically tunable chiral plasmonic guest-host cellulose films weaved with long-range ordered silver nanowires. ACS Appl Mater Interfaces.

[44] Lv J, Ding D, Yang X, Hou K, Miao X, Wang D (2019). Biomimetic chiral photonic crystals. Angew Chem Int Ed Engl.

[45] Chen W, Bian A, Agarwal A, Liu L, Shen H, Wang L (2009). Nanoparticle superstructures made by polymerase chain reaction: Collective interactions of nanoparticles and a new principle for chiral materials. Nano Lett.

[46] Karimullah AS, Jack C, Tullius R, Rotello VM, Cooke G, Gadegaard N (2015). Disposable plasmonics: Plastic templated plasmonic metamaterials with tunable chirality. Adv Mater.

[47] Fu H, Hu O, Fan Y, Hu Y, Huang J, Wang Z (2019). Rational design of an “on-off-on” fluorescent assay for chiral amino acids based on quantum dots and nanoporphyrin. Sens Actuators B Chem.

[48] Liu Y, Li Y, Jeong S, Wang Y, Chen J, Ye X (2020). Colloidal synthesis of nanohelices *via* bilayer lattice misfit. J Am Chem Soc.

[49] Saito K, Tatsuma T (2018). Chiral plasmonic nanostructures fabricated by circularly polarized light. Nano Lett.

[50] Wei X, Liu J, Xia GJ, Deng J, Sun P, Chruma JJ (2020). Enantioselective photoinduced cyclodimerization of a prochiral anthracene derivative adsorbed on helical metal nanostructures. Nat Chem.

[51] Liu J, Yang L, Huang Z (2016). Chiroptically active plasmonic nanoparticles having hidden helicity and reversible aqueous solvent effect on chiroptical activity. Small.

[52] Liu J, Yang L, Zhang H, Wang J, Huang Z (2017). Ultraviolet-visible chiroptical activity of aluminum nanostructures. Small.

[53] Yang L, Kwan CS, Zhang L, Li X, Han Y, Leung KCF (2019). Chiral nanoparticle-induced enantioselective amplification of molecular optical activity. Adv Funct Mater.

[54] Yang L, Liu J, Sun P, Ni Z, Ma Y, Huang Z (2020). Chiral ligand-free, optically active nanoparticles inherently composed of chiral lattices at the atomic scale. Small.

[55] Ni Z, Zhu Y, Liu J, Yang L, Sun P, Gu M (2020). Extension of compositional space to the ternary in alloy chiral nanoparticles through galvanic replacement reactions. Adv Sci.

[56] Koch SW, Kira M, Khitrova G, Gibbs HM (2006). Semiconductor excitons in new light. Nat Mater.

[57] Nozik AJ (2008). Multiple exciton generation in semiconductor quantum dots. Chem Phys Lett.

[58] Hartland GV (2011). Optical studies of dynamics in noble metal nanostructures. Chem Rev.

[59] Li M, Cushing SK, Wu N (2015). Plasmon-enhanced optical sensors: A review. Analyst.

[60] Mulvaney P (1996). Surface plasmon spectroscopy of nanosized metal particles. Langmuir.

[61] Mayer KM, Hafner JH (2011). Localized surface plasmon resonance sensors. Chem Rev.

[62] Eustis S, El-Sayed MA (2006). Determination of the aspect ratio statistical distribution of gold nanorods in solution from a theoretical fit of the observed inhomogeneously broadened longitudinal plasmon resonance absorption spectrum. J Appl Phys.

[63] Link S, Mohamed MB, El-Sayed MA (2005). Simulation of the optical absorption spectra of gold nanorods as a function of their aspect ratio and the effect of the medium dielectric constant. J Phys Chem B.

[64] Gautier C, Bürgi T (2009). Chiral gold nanoparticles. Chemphyschem.

[65] Kitaev V (2008). Chiral nanoscale building blocks—from understanding to applications. J Mater Chem.

[66] Xia Y, Zhou Y, Tang Z (2011). Chiral inorganic nanoparticles: Origin, optical properties and bioapplications. Nanoscale.

[67] Noguez C, Sánchez-Castillo A, Hidalgo F (2011). Role of morphology in the enhanced optical activity of ligand-protected metal nanoparticles. J Phys Chem Lett.

[68] Goldsmith MR, George CB, Zuber G, Naaman R, Waldeck DH, Wipf P (2006). The chiroptical signature of achiral metal clusters induced by dissymmetric adsorbates. Phys Chem Chem Phys.

[69] Govorov AO, Fan Z, Hernandez P, Slocik JM, Naik RR (2010). Theory of circular dichroism of nanomaterials comprising chiral molecules and nanocrystals: Plasmon enhancement, dipole interactions, and dielectric effects. Nano Lett.

[70] Govorov AO (2011). Plasmon-induced circular dichroism of a chiral molecule in the vicinity of metal nanocrystals. Application to various geometries. J Phys Chem C.

[71] Govorov AO, Gun'ko YK, Slocik JM, Gérard VA, Fan Z, Naik RR (2011). Chiral nanoparticle assemblies: Circular dichroism, plasmonic interactions, and exciton effects. J Mater Chem.

[72] Shen X, Asenjo-Garcia A, Liu Q, Jiang Q, García de Abajo FJ, Liu N (2013). Three-dimensional plasmonic chiral tetramers assembled by DNA origami. Nano Lett.

[73] Rafiei Miandashti A, Khosravi Khorashad L, Kordesch ME, Govorov AO, Richardson HH (2020). Experimental and theoretical observation of photothermal chirality in gold nanoparticle helicoids. ACS Nano.

[74] Kong XT, Khosravi Khorashad L, Wang Z, Govorov AO (2018). Photothermal circular dichroism induced by plasmon resonances in chiral metamaterial absorbers and bolometers. Nano Lett.

[75] Longhi G, Castiglioni E, Koshoubu J, Mazzeo G, Abbate S (2016). Circularly polarized luminescence: A review of experimental and theoretical aspects. Chirality.

[76] Berova N, Polavarapu PL, Nakanishi K, Woody RW (2012). Comprehensive chiroptical spectroscopy, instrumentation, methodologies, and theoretical simulations (volume 1). New Jersey, USA: John Wiley & Sons, Inc.

[77] Arrico L, Bari LD, Zinna F (2021). Quantifying the overall efficiency of circularly polarized emitters. Chemistry.

[78] Wang Y, Xia Y (2020). Near-infrared optically active Cu_2-x_S nanocrystals: Sacrificial template-ligand exchange integration fabrication and chirality dependent autophagy effects. J Mater Chem B.

[79] Yang F, Gao G, Wang J, Chen R, Zhu W, Wang L (2019). Chiral β-HgS quantum dots: Aqueous synthesis, optical properties and cytocompatibility. J Colloid Interface Sci.

[80] Miao J, Cai Y, Shao Y, Yang G, Huang H, Shang Z (2021). Multiple cell death pathways triggered by temperature-mediated synergistic effect derived from chiral phototheranostic ablation nanoagents. Appl Mater Today.

[81] Mang TS (2004). Lasers and light sources for PDT: Past, present and future. Photodiagnosis Photodyn Ther.

[82] Chatterjee DK, Fong LS, Zhang Y (2008). Nanoparticles in photodynamic therapy: An emerging paradigm. Adv Drug Deliv Rev.

[83] Allison RR, Downie GH, Cuenca R, Hu XH, Childs CJH, Sibata CH (2004). Photosensitizers in clinical PDT. Photodiagnosis Photodyn Ther.

[84] Cheng H, Kamegawa T, Mori K, Yamashita H (2014). Surfactant-free nonaqueous synthesis of plasmonic molybdenum oxide nanosheets with enhanced catalytic activity for hydrogen generation from ammonia borane under visible light. Angew Chem Int Ed Engl.

[85] He T, Qiu X, Li J, Pang G, Wu Z, Cheng J (2019). Water-soluble chiral CdSe/CdS dot/rod nanocrystals for two-photon fluorescence lifetime imaging and photodynamic therapy. Nanoscale.

[86] Sun M, Xu L, Bahng JH, Kuang H, Alben S, Kotov NA (2017). Intracellular localization of nanoparticle dimers by chirality reversal. Nat Commun.

[87] Xiao S, Lu Y, Feng M, Dong M, Cao Z, Zhang X (2020). Multifunctional FeS_2_ theranostic nanoparticles for photothermal-enhanced chemodynamic/photodynamic cancer therapy and photoacoustic imaging. Chem Eng J.

[88] Jia TT, Li BJ, Yang G, Hua Y, Liu JQ, Ma W (2021). Enantiomeric alkynyl-protected Au_10_ clusters with chirality-dependent radiotherapy enhancing effects. Nano Today.

[89] Gao F, Sun M, Ma W, Wu X, Liu L, Kuang H (2017). A singlet oxygen generating agent by chirality-dependent plasmonic shell-satellite nanoassembly. Adv Mater.

[90] Wang J, Wu X, Ma W, Xu C (2020). Chiral AuCuAu heterogeneous nanorods with tailored optical activity. Adv Funct Mater.

[91] Fass L (2008). Imaging and cancer: A review. Mol Oncol.

[92] Weber J, Beard PC, Bohndiek SE (2016). Contrast agents for molecular photoacoustic imaging. Nat Methods.

[93] Soto C (2003). Unfolding the role of protein misfolding in neurodegenerative diseases. Nat Rev Neurosci.

[94] Barnham KJ, Masters CL, Bush AI (2004). Neurodegenerative diseases and oxidative stress. Nat Rev Drug Discov.

[95] Saraiva C, Praça C, Ferreira R, Santos T, Ferreira L, Bernardino L (2016). Nanoparticle-mediated brain drug delivery: Overcoming blood-brain barrier to treat neurodegenerative diseases. J Control Release.

[96] Hao C, Qu A, Xu L, Sun M, Zhang H, Xu C (2019). Chiral molecule-mediated porous Cu_x_O nanoparticle clusters with antioxidation activity for ameliorating parkinson's disease. J Am Chem Soc.

[97] Hou K, Zhao J, Wang H, Li B, Li K, Shi X (2020). Chiral gold nanoparticles enantioselectively rescue memory deficits in a mouse model of alzheimer's disease. Nat Commun.

[98] Zhang H, Hao C, Qu A, Sun M, Xu L, Xu C (2020). Light-induced chiral iron copper selenide nanoparticles prevent beta-amyloidopathy *in vivo*. Angew Chem Int Ed Engl.

[99] Liu Q, Zhang A, Wang R, Zhang Q, Cui D (2021). A review on metal- and metal oxide-based nanozymes: Properties, mechanisms, and applications. Nanomicro Lett.

[100] Zhang H, Li S, Qu A, Hao C, Sun M, Xu L (2020). Engineering of chiral nanomaterials for biomimetic catalysis. Chem Sci.

[101] Li F, Li S, Guo X, Dong Y, Yao C, Liu Y (2020). Chiral carbon dots mimicking topoisomerase Ⅰ to mediate the topological rearrangement of supercoiled DNA enantioselectively. Angew Chem Int Ed Engl.

[102] Gao H (2014). Probing mechanical principles of cell-nanomaterial interactions. J Mech Phys Solids.

[103] Tsoi KM, MacParland SA, Ma XZ, Spetzler VN, Echeverri J, Ouyang B (2016). Mechanism of hard-nanomaterial clearance by the liver. Nat Mater.

[104] Zhang L, Wang T, Shen Z, Liu M (2016). Chiral nanoarchitectonics: Towards the design, self-assembly, and function of nanoscale chiral twists and helices. Adv Mater.

[105] Zhang M, Qing G, Sun T (2012). Chiral biointerface materials. Chem Soc Rev.

[106] Sun M, Hao T, Li X, Qu A, Xu L, Hao C (2018). Direct observation of selective autophagy induction in cells and tissues by self-assembled chiral nanodevice. Nat Commun.

[107] Li Y, Zhou Y, Wang HY, Perrett S, Zhao Y, Tang Z (2011). Chirality of glutathione surface coating affects the cytotoxicity of quantum dots. Angew Chem Int Ed Engl.

[108] Lu M, Qu A, Li S, Sun M, Xu L, Kuang H (2020). Mitochondria-targeting plasmonic spiky nanorods increase the elimination of aging cells *in vivo*. Angew Chem Int Ed Engl.

[109] Li S, Sun M, Hao C, Qu A, Wu X, Xu L (2020). Chiral Cu_x_Co_y_S nanoparticles under magnetic field and NIR light to eliminate senescent cells. Angew Chem Int Ed Engl.

[110] Huang Y, Fu Y, Li M, Jiang D, Kutyreff CJ, Engle JW (2020). Chirality-driven transportation and oxidation prevention by chiral selenium nanoparticles. Angew Chem Int Ed Engl.

[111] Deng J, Zheng H, Zheng X, Yao M, Li Z, Gao C (2016). Gold nanoparticles with surface-anchored chiral poly(acryloyl-l(d)-valine) induce differential response on mesenchymal stem cell osteogenesis. Nano Res.

[112] Zhao X, Xu L, Sun M, Ma W, Wu X, Xu C (2017). Tuning the interactions between chiral plasmonic films and living cells. Nat Commun.

[113] Kumar J, Eraa H, López-Martínez E, Claes N, Martín VF, Solís DM (2018). Detection of amyloid fibrils in parkinson's disease using plasmonic chirality. Proc Natl Acad Sci U S A.

[114] Martynenko IV, Kuznetsova VA, Litvinov IK, Orlova AO, Maslov VG, Fedorov AV (2016). Enantioselective cellular uptake of chiral semiconductor nanocrystals. Nanotechnology.

[115] Ngamdee K, Ngeontae W (2018). Circular dichroism glucose biosensor based on chiral cadmium sulfide quantum dots. Sens Actuators B Chem.

[116] Hao C, Gao R, Li Y, Xu L, Sun M, Xu C (2019). Chiral semiconductor nanoparticles for protein catalysis and profiling. Angew Chem Int Ed Engl.

[117] Tedsana W, Tuntulani T, Ngeontae W (2015). A circular dichroism sensor for Ni^2+^ and Co^2+^ based on L-cysteine capped cadmium sulfide quantum dots. Anal Chim Acta.

[118] Masteri-Farahani M, Khademabbasi K, Mollatayefeh N, Schneider R (2018). L- and D-cysteine functionalized CdS quantum dots as nanosensors for detection of L-morphine and D-methamphetamine. J Nanostruct.

[119] Ghasemi F, Hormozi-Nezhad MR, Mahmoudi M (2017). Time-resolved visual chiral discrimination of cysteine using unmodified CdTe quantum dots. Sci Rep.

[120] Gao F, Ma S, Xiao X, Hu Y, Zhao D, He Z (2016). Sensing tyrosine enantiomers by using chiral CdSe/CdS quantum dots capped with N-acetyl-l-cysteine. Talanta.

[121] Song L, Wang S, Kotov NA, Xia Y (2012). Nonexclusive fluorescent sensing for L/D enantiomers enabled by dynamic nanoparticle-nanorod assemblies. Anal Chem.

[122] Sianglam P, Kulchat S, Tuntulani T, Ngeontae W (2017). A circular dichroism sensor for selective detection of Cd^2+^ and S^2-^ based on the *in-situ* generation of chiral CdS quantum dots. Spectrochim Acta A Mol Biomol Spectrosc.

[123] Noipa T, Ngamdee K, Tuntulani T, Ngeontae W (2014). Cysteamine CdS quantum dots decorated with Fe^3+^ as a fluorescence sensor for the detection of PPi. Spectrochim Acta A Mol Biomol Spectrosc.

[124] Ngamdee K, Chaiendoo K, Saiyasombat C, Busayaporn W, Ittisanronnachai S, Promarak V (2018). Highly selective circular dichroism sensor based on D-penicillamine/cysteaminecadmium sulfide quantum dots for copper (Ⅱ) ion detection. Spectrochim Acta A Mol Biomol Spectrosc.

[125] Guo Y, Zeng X, Yuan H, Huang Y, Zhao Y, Wu H (2017). Chiral recognition of phenylglycinol enantiomers based on N-acetyl-L-cysteine capped CdTe quantum dots in the presence of Ag. Spectrochim Acta A Mol Biomol Spectrosc.

[126] Wang X, Hao J, Cheng J, Li J, Miao J, Li R (2019). Chiral CdSe nanoplatelets as an ultrasensitive probe for lead ion sensing. Nanoscale.

[127] Durán GM, Abellán C, Contento AM, Ríos Á (2017). Discrimination of penicillamine enantiomers using β-cyclodextrin modified CdSe/ZnS quantum dots. Microchim Acta.

[128] Freeman R, Finder T, Bahshi L, Willner I (2009). Beta-cyclodextrin-modified CdSe/ZnS quantum dots for sensing and chiroselective analysis. Nano Lett.

[129] Han C, Li H (2008). Chiral recognition of amino acids based on cyclodextrin-capped quantum dots. Small.

[130] Delgado-Pérez T, Bouchet LM, delaGuardia M, Galian RE, Pérez-Prieto J (2013). Sensing chiral drugs by using CdSe/ZnS nanoparticles capped with N-acetyl-L-cysteine methyl ester. Eur J Chem.

[131] Carrillo-Carrión C, Cárdenas S, Simonet BM, Valcárcel M (2009). Selective quantification of carnitine enantiomers using chiral cysteine-capped CdSe(ZnS) quantum dots. Anal Chem.

[132] Wei Y, Li H, Hao H, Chen Y, Dong C, Wang G (2014). β-Cyclodextrin functionalized Mn-doped ZnS quantum dots for the chiral sensing of tryptophan enantiomers. Polym Chem.

[133] Lin J, Huang B, Dai Y, Wei J, Chen Y (2018). Chiral ZnO nanoparticles for detection of dopamine. Mater Sci Eng C Mater Biol Appl.

[134] Ahmed SR, Neethirajan S (2018). Chiral MoS_2_ quantum dots: Dual-mode detection approaches for avian influenza viruses. Glob Chall.

[135] Hao J, Li Y, Xu X, Zhao F, Pan R, Li J (2020). Metal-to-Ligand Charge Transfer Chirality Sensing of D-Glucose Assisted with GOX-Based Enzymatic Reaction. Adv Mater Technol.

[136] Wang X, Wang Q, Chen Y, Li J, Chen R (2020). Metal-to-ligand charge transfer chirality-based sensing of mercury ions. Photonics Res.

[137] Tang L, Li S, Xu L, Ma W, Kuang H, Wang L (2015). Chirality-based Au@Ag Nanorod Dimers Sensor for Ultrasensitive PSA Detection. ACS Appl Mater Interfaces.

[138] Zhao Y, Yang Y, Zhao J, Weng P, Pang Q, Song Q (2016). Dynamic chiral nanoparticle assemblies and specific chiroplasmonic analysis of cancer cells. Adv Mater.

[139] Ma W, Sun M, Fu P, Li S, Xu L, Kuang H (2017). A chiral-nanoassemblies-enabled strategy for simultaneously profiling surface glycoprotein and microrna in living cells. Adv Mater.

[140] Meng D, Ma W, Wu X, Xu C, Kuang H (2020). DNA-driven two-layer core-satellite gold nanostructures for ultrasensitive microrna detection in living cells. Small.

[141] Song G, Zhou F, Xu C, Li B (2017). A universal strategy for visual chiral recognition of α-amino acids with l-tartaric acid-capped gold nanoparticles as colorimetric probes. Analyst.

[142] Wang Y, Zhou X, Liu Q, Jin Y, Li B (2020). Gold nanorods as colorimetric probes for naked-eye recognition of carnitine enantiomers. Gold Bull.

[143] Wang Y, Zhou X, Xu C, Jin Y, Li B (2018). Gold nanorods as visual sensing platform for chiral recognition with naked eyes. Sci Rep.

[144] Ma W, Kuang H, Xu L, Ding L, Xu C, Wang L (2013). Attomolar DNA detection with chiral nanorod assemblies. Nat Commun.

[145] Zhu F, Li X, Li Y, Yan M, Liu S (2015). Enantioselective circular dichroism sensing of cysteine and glutathione with gold nanorods. Anal Chem.

[146] Abbasi S, Khani H (2017). Highly selective and sensitive method for Cu^2+^ detection based on chiroptical activity of L-Cysteine mediated Au nanorod assemblies. Spectrochim Acta A Mol Biomol Spectrosc.

[147] Wang RY, Wang P, Liu Y, Zhao W, Zhai D, Hong X (2014). Experimental observation of giant chiroptical amplification of small chiral molecules by gold nanosphere clusters. J Phys Chem C.

[148] Kang YJ, Oh JW, Kim YR, Kim JS, Kim H (2010). Chiral gold nanoparticle-based electrochemical sensor for enantioselective recognition of 3,4-dihydroxyphenylalanine. Chem Commun.

[149] Li R, Zhu C, Wang L, Zhang X, Ji Y (2018). A new nanosensor for the chiral recognition of cysteine enantiomers based on gold nanorods. New J Chem.

[150] Kuang H, Chen X, Hao C, Ma W, Xu L, Xu C (2013). Immuno-driven plasmonic oligomer sensor for the ultrasensitive detection of antibiotics. RSC Adv.

[151] Kuang H, Yin H, Liu L, Xu L, Xu C (2014). Asymmetric plasmonic aptasensor for sensitive detection of bisphenol A. ACS Appl Mater Interfaces.

[152] Xu Z, Xu L, Liz-Marzán LM, Ma W, Kotov NA, Wang L (2013). Sensitive detection of silver ions based on chiroplasmonic assemblies of nanoparticles. Adv Opt Mater.

[153] Liu Y, Wei M, Zhang L, Wei W, Zhang Y, Liu S (2015). Evaluation of DNA methyltransferase activity and inhibition *via* chiroplasmonic assemblies of gold nanoparticles. Chem Comm.

[154] Liu Y, Wei M, Zhang L, Zhang Y, Wei W, Yin L (2016). Chiroplasmonic assemblies of gold nanoparticles for ultrasensitive detection of 8-hydroxy-2'-deoxyguanosine in human serum sample. Anal Chem.

[155] Fu P, Sun M, Xu L, Wu X, Liu L, Kuang H (2016). A self-assembled chiral-aptasensor for ATP activity detection. Nanoscale.

[156] Xu L, Xu Z, Ma W, Liu L, Wang L, Kuang H (2013). Highly selective recognition and ultrasensitive quantification of enantiomers. J Mater Chem B.

[157] Zhao H, Bian S, Yang Y, Wu X (2017). Chiroplasmonic assemblies of gold nanoparticles as a novel method for sensitive detection of alpha-fetoprotein. Mikrochim Acta.

[158] Zhou X, Xu L, Zhu Y, Wei M, Hua K, Wang L (2012). Chirality based sensor for bisphenol a detection. Chem Commun.

[159] Wu X, Xu L, Ma W, Liu L, Kuang H, Kotov NA (2016). Propeller-like nanorod-upconversion nanoparticle assemblies with intense chiroptical activity and luminescence enhancement in aqueous phase. Adv Mater.

[160] Cai J, Hao C, Sun M, Ma W, Xu C, Kuang H (2018). Chiral shell core-satellite nanostructures for ultrasensitive detection of mycotoxin. Small.

[161] Zhang Y, Liu J, Da L, Xing D, Yang W (2016). Self-assembled core-satellite gold nanoparticle networks for ultrasensitive detection of chiral molecules by recognition tunneling. ACS Nano.

[162] Yang F, Kong N, Conlan XA, Wang H, Barrow CJ, Yan F (2017). Electrochemical evidences of chiral molecule recognition using L/D-cysteine modified gold electrodes. Electrochim Acta.

[163] Yan W, Xu L, Ma W, Liu L, Wang L, Kuang H (2014). Pyramidal sensor platform with reversible chiroptical signals for DNA detection. Small.

[164] Zhao Y, Xu L, Ma W, Wang L, Kuang H, Xu C (2014). Shell-engineered chiroplasmonic assemblies of nanoparticles for zeptomolar DNA detection. Nano Lett.

[165] Zhu Y, Xu L, Ma W, Zhou X, Hua K, Wang L (2012). A one-step homogeneous plasmonic circular dichroism detection of aqueous mercury ions using nucleic acid functionalized gold nanorods. Chem Commun.

[166] Si L, Xu L, Ma W, Wu X, Sun M, Hua K (2016). Dual-mode ultrasensitive quantification of microrna in living cells by chiroplasmonic nanopyramids self-assembled from gold and upconversion nanoparticles. J Am Chem Soc.

[167] Taron W, Jamnongkan W, Techasen A, Phetcharaburanin J, Namwat N, Sithithaworn P (2020). AuNPs-LISA, an efficient detection assay for Opisthorchis viverrini (Ov) antigen in urine. Talanta.

[168] Song G, Xu C, Li B (2015). Visual chiral recognition of mandelic acid enantiomers with L-tartaric acid-capped gold nanoparticles as colorimetric probes. Sens Actuators B Chem.

[169] Ma W, Xu L, Wang L, Xu C, Kuang H (2019). Chirality-based biosensors. Adv Funct Mater.

[170] Wu X, Xu L, Liu L, Ma W, Yin H, Kuang H (2013). Unexpected chirality of nanoparticle dimers and ultrasensitive chiroplasmonic bioanalysis. J Am Chem Soc.

[171] Zhao X, Wu X, Xu L, Ma W, Kuang H, Wang L (2015). Building heterogeneous core-satellite chiral assemblies for ultrasensitive toxin detection. Biosens Bioelectron.

[172] Bao ZY, Zhang W, Zhang YL, He J, Dai J, Yeung CT (2017). Interband absorption enhanced optical activity in discrete Au@Ag core-shell nanocuboids: Probing extended helical conformation of chemisorbed cysteine molecules. Angew Chem Int Ed Engl.

[173] Su H, Zheng Q, Li H (2012). Colorimetric detection and separation of chiral tyrosine based on N-acetyl-L-cysteine modified gold nanoparticles. J Mater Chem.

[174] Shukla N, Bartel MA, Gellman AJ (2010). Enantioselective separation on chiral Au nanoparticles. J Am Chem Soc.

[175] Wang Y, Yin X, Shi M, Li W, Lei Z, Kong J (2006). Probing chiral amino acids at sub-picomolar level based on bovine serum albumin enantioselective films coupled with silver-enhanced gold nanoparticles. Talanta.

[176] Barron LD, Gadegaard N, Kadodwala M, Hendry E, Carpy T, Johnston J (2010). Ultrasensitive detection and characterization of biomolecules using superchiral fields. Nat Nanotechnol.

[177] Ye ZBC (2011). Colorimetric chiral recognition of enantiomers using the nucleotide-capped silver nanoparticles. Anal Chem.

[178] Sun Y, Zhang L, Li H (2012). Chiral colorimetric recognition of amino acids based on silver nanoparticle clusters. New J Chem.

[179] Kuang H, Yin H, Xing C, Xu C (2013). A sensitive dnazyme-based chiral sensor for lead detection. Materials.

[180] Sun X, Kong H, Zhou Q, Tsunega S, Liu X, Yang H (2020). Chiral plasmonic nanoparticle assisted raman enantioselective recognition. Anal Chem.

[181] Liu Z, Ai J, Kumar P, You E, Zhou X, Liu X (2020). Enantiomeric discrimination by surface-enhanced raman scattering-chiral anisotropy of chiral nanostructured gold films. Angew Chem Int Ed Engl.

[182] Chen Y, Xu J, Cui C, Guo D, Fu Y (2015). The application of L-tryptophan functionalized graphene-supported platinum nanoparticles for chiral recogniton of dopa enantiomers. New J Chem.

[183] Ahmed SR, Nagy E, Neethirajan S (2017). Self-assembled star-shaped chiroplasmonic gold nanoparticles for an ultrasensitive chiro-immunosensor for viruses. RSC Adv.

[184] Tang H, Li Q, Yan W, Jiang X (2021). Reversing the chirality of surface ligands can improve the biosafety and pharmacokinetics of cationic gold nanoclusters. Angew Chem Int Ed Engl.

[185] Yeom J, Guimaraes PPG, Ahn HM, Jung BK, Hu Q, McHugh K (2020). Chiral supraparticles for controllable nanomedicine. Adv Mater.

[186] Zhang NN, Sun HR, Liu S, Xing YC, Lu J, Peng F (2021). Gold nanoparticle enantiomers and their chiral-morphology dependence of cellular uptake. CCS Chem.

[187] Visheratina A, Kotov NA (2020). Inorganic nanostructures with strong chiroptical activity. CCS Chem.

[188] Poon W, Zhang YN, Ouyang B, Kingston BR, Wu JLY, Wilhelm S (2019). Elimination pathways of nanoparticles. ACS Nano.

[189] Barron LD (2008). Chirality and life. Space Sci Rev.

[190] Lough WJ, Wainer IW (2002). Chirality in the natural and applied sciences. Oxford: Blackwell.

[191] Ahn HY, Yoo S, Cho NH, Kim RM, Kim H, Huh JH (2019). Bioinspired toolkit based on intermolecular encoder toward evolutionary 4D chiral plasmonic materials. Acc Chem Res.

[192] Li J, Li J, Liu R, Tu Y, Li Y, Cheng J (2020). Autonomous discovery of optically active chiral inorganic perovskite nanocrystals through an intelligent cloud lab. Nat Commun.

[193] Feng W, Kim J, Wang X, Calcaterra H, Qu Z, Meshi L (2017). Assembly of mesoscale helices with near-unity enantiomeric excess and light-matter interactions for chiral semiconductors. Sci Adv.

[194] Roche C, Sun H, Prendergast M, Leowanawat P, Partridge B, Heiney P (2014). Homochiral columns constructed by chiral self-sorting during supramolecular helical organization of hat-shaped molecules. J Am Chem Soc.

[195] Song M, Tong L, Liu S, Zhang Y, Dong J, Ji Y (2021). Nonlinear amplification of chirality in self-assembled plasmonic nanostructures. ACS Nano.

[196] Srivastava S, Santos A, Critchley K, Kim K, Podsiadlo P, Sun K (2010). Light-controlled self-assembly of semiconductor nanoparticles into twisted ribbons. Science.

[197] Zhou M, Sang Y, Jin X, Chen S, Guo J, Duan P (2021). Steering nanohelix and upconverted circularly polarized luminescence by using completely achiral components. ACS Nano.

